# Aerobic methanotrophic communities at the Red Sea brine-seawater interface

**DOI:** 10.3389/fmicb.2014.00487

**Published:** 2014-09-23

**Authors:** Rehab Z. Abdallah, Mustafa Adel, Amged Ouf, Ahmed Sayed, Mohamed A. Ghazy, Intikhab Alam, Magbubah Essack, Feras F. Lafi, Vladimir B. Bajic, Hamza El-Dorry, Rania Siam

**Affiliations:** ^1^Biotechnology Graduate Program, American University in CairoCairo, Egypt; ^2^Department of Biology, American University in CairoCairo, Egypt; ^3^Computer, Electrical and Mathematical Sciences and Engineering Division, Computational Bioscience Research Center, King Abdullah University of Science and TechnologyThuwal, Saudi Arabia; ^4^YJ-Science and Technology Research Center, American University in CairoCairo, Egypt

**Keywords:** brine-seawater interfaces, aerobic methanotrophs, *pmoA*, 16S rRNA gene, Red Sea

## Abstract

The central rift of the Red Sea contains 25 brine pools with different physicochemical conditions, dictating the diversity and abundance of the microbial community. Three of these pools, the Atlantis II, Kebrit and Discovery Deeps, are uniquely characterized by a high concentration of hydrocarbons. The brine-seawater interface, described as an anoxic-oxic (brine-seawater) boundary, is characterized by a high methane concentration, thus favoring aerobic methane oxidation. The current study analyzed the aerobic free–living methane-oxidizing bacterial communities that potentially contribute to methane oxidation at the brine-seawater interfaces of the three aforementioned brine pools, using metagenomic pyrosequencing, 16S rRNA pyrotags and *pmoA* library constructs. The sequencing of 16S rRNA pyrotags revealed that these interfaces are characterized by high microbial community diversity. Signatures of aerobic methane-oxidizing bacteria were detected in the Atlantis II Interface (ATII-I) and the Kebrit Deep Upper (KB-U) and Lower (KB-L) brine-seawater interfaces. Through phylogenetic analysis of *pmoA*, we further demonstrated that the ATII-I aerobic methanotroph community is highly diverse. We propose four ATII-I *pmoA* clusters. Most importantly, cluster 2 groups with marine methane seep methanotrophs, and cluster 4 represent a unique lineage of an uncultured bacterium with divergent alkane monooxygenases. Moreover, non-metric multidimensional scaling (NMDS) based on the ordination of putative enzymes involved in methane metabolism showed that the Kebrit interface layers were distinct from the ATII-I and DD-I brine-seawater interfaces.

## Introduction

The Red Sea is a 450,000 square kilometer inlet of the Indian Ocean bordered by Egypt, Eritrea, Sudan and Djibouti to the west, Saudi Arabia and Yemen to the east and Egypt, Israel, Jordan and Saudi Arabia to the north. It has been described as an ocean *in statu nascendi* (Antunes et al., 2011a). The divergent movement of the African and Arabian tectonic plates exposed the hot mantle rock of the asthenosphere, causing a geothermal solution to be injected through the Earth's crust and mix with Red Sea seawater to form deep-sea brine pools (Oudin et al., 1984; Oudin and Thisse, 1988). One of the characteristics of the Red Sea is the presence of deep brines (Antunes et al., 2011b). The central rift of the Red Sea contains ~25 brine pools (Hartmann et al., 1998). Due to their distinctive physical and geochemical properties, deep-sea brine pools such as the Atlantis II Deep, Discovery Deep and Kebrit Deep are of particular interest. The Atlantis II Deep is known for its harsh and extreme environment, showing a temperature of ~67.1°C and a salinity of 252 psu (Swift et al., 2012). The Discovery Deep is located southwest of Atlantis II and exhibits a temperature of ~45°C and a salinity of 100 psu (Swift et al., 2012). On the other hand, the Kebrit Deep displays an ambient water temperature (22–33°C) but is characterized by an elevated concentration of H_2_S (ranging from 12 to 14 mg sulfur/l) (Hartmann et al., 1998; Stoffers et al., 1998). Compared with the surface water methane concentration (40 nl/l–1.8 nmol/l), these brine pools are also characterized by a high concentration of hydrocarbons, including methane, which seeps from the brine into the overlaying brine-seawater interface (Faber et al., 1998; Hartmann et al., 1998; Schmidt et al., 2003). The Kebrit Deep exhibits the highest concentration, which reaches a maximum of 476.2 mmol/l, followed by the Atlantis II Deep and Discovery Deep, with concentrations of 5.5 mmol/l and 0.81 mmol/l, respectively (Schmidt et al., 2003).

The overlaying brine-seawater interface is an aerobic methane-rich layer; therefore, it represents a favorable environment for aerobic methane oxidation (Faber et al., 1998; Schmidt et al., 2003). The methane concentration in interface layers can be as high as 276.2 mmol/l in the Kebrit Deep, 0.983 mmol/l in the Atlantis II Deep, and 0.81 mmol/l in the Discovery Deep (Schmidt et al., 2003). Moreover, carbon isotope analyses have suggested the occurrence of aerobic methane oxidation in the brine-seawater interface layers of the Atlantis II, Discovery and Kebrit Deeps (Faber et al., 1998; Schmidt et al., 2003). Positive shifts in 13C isotope levels, with δ^13^C–CH values of +5.7‰ PDB (Pee Dee Belemnite) and +26.5‰ were reported in the Atlantis II and Discovery Deep interfaces, respectively (Schmidt et al., 2003).

Aerobic methanotrophic bacteria have been discussed in the scientific literature following the isolation of the first aerobic methanotroph (*Bacillus methanicus*) by Söhngen (1906), Hanson and Hanson (1996). To date, the only known facultative methanotrophs are type II methanotrophs, belonging to the α–proteobacteria class, while type I methanotrophs belong to the γ–proteobacteria class (Hanson and Hanson, 1996; McDonald et al., 2008; Murrell, 2010). All aerobic methanotrophs possess the particulate methane monooxygenase (pMMO) gene, except for facultative methanotrophs of the *Methylocella* genus and the obligate methanotroph *Methyloferula stellate* (Dedysh et al., 2005; Vorobev et al., 2011). Facultative methanotrophs also possess the soluble methane monooxygenase (sMMO) gene (Dedysh et al., 2005; Dunfield et al., 2010; Belova et al., 2011; Im et al., 2011). The filamentous methane oxidizers, *Crenothrix polyspora* and *Clonothrix fusca*, were shown to be γ–proteobacteria that are closely related to methanotrophs (Stoecker et al., 2006; Vigliotta et al., 2007). However, Proteobacteria is not the only bacterial phylum that includes aerobic methanotrophs, as a class of aerobic methanotrophs belonging to the phylum Verrucomicrobia was found to thrive in highly acidic environments (Dunfield et al., 2007; Pol et al., 2007; Islam et al., 2008).

It is important to improve our knowledge of methane-oxidizing bacteria or methanotrophic communities, as they contribute to our understanding of methane cycling in the environment. Several studies have discussed the presence of aerobic methanotrophs in a wide range of marine habitats, including water column and sediment samples rich in methane collected from hydrothermal vents and hydrocarbon seeps (Wang et al., 2004; Nercessian et al., 2005; Yan et al., 2006; Tavormina et al., 2008, 2010; Kato et al., 2009; Moussard et al., 2009; Reed et al., 2009; Wasmund et al., 2009; Redmond et al., 2010). Studies examining the diversity of aerobic methanotrophic bacteria in the marine environment have mainly been carried out using the *pmoA* gene, encoding a 27-kDa polypeptide subunit of particulate methane monooxygenase (pMMO), or the 16S rRNA gene. Novel marine-specific aerobic methanotrophs (OPU1, OPU3, and Group X) were discovered recently and have been suggested to be the major group involved in aerobic methane oxidation in oceanic systems (Elsaied et al., 2004; Tavormina et al., 2008, 2010; Wasmund et al., 2009). OPU1 and OPU3 represent a lineage that is distantly related to the type I methanotrophs (specifically the *Methylocladum* and *Methylococcus* genera) (Elsaied et al., 2004; Tavormina et al., 2008, 2010; Wasmund et al., 2009). However, Group X represents an out-group of both type I and type II methanotrophs (Tavormina et al., 2008, 2010; Wasmund et al., 2009). It is worth noting that 16S rRNA sequences were not recovered from the samples of these groups of methanotrophs (Tavormina et al., 2008; Wasmund et al., 2009). Except recently, candidate 16S rRNA genes for these groups were identified in the Costa Rica convergent margin oxygen minimum zone (Tavormina et al., 2013). These lineages have been reported to be different from aerobic methanotrophs that have been identified in marine sediments (Tavormina et al., 2008, 2010; Wasmund et al., 2009). Thus, far, methane oxidation has only been studied in the three geochemically different brine pools in the Red Sea mentioned above (the Atlantis II Deep, Discovery Deep and Kebrit Deep) through carbon isotopes analyses, without any molecular and biological associations being obtained (Faber et al., 1998; Schmidt et al., 2003).

In this study, we investigate the aerobic methanotrophic bacterial communities that contribute to methane oxidation at the brine-seawater interface in these Red Sea brine pools. Because the brine-seawater interface layers exhibit differential levels of methane/oxygen, the abundance/diversity of the aerobic methanotrophic community should vary in the different seawater layers. In particular, the presence of type I methanotrophs, which are known to dominate marine environments, is expected (Nercessian et al., 2005; Yan et al., 2006; Tavormina et al., 2008; Reed et al., 2009). We adopted a comprehensive approach based on the shotgun pyrosequencing of metagenomic sequencing libraries, in addition to the use of 16S rRNA pyrotags and *pmoA* library analyses.

## Materials and methods

### Sample collection

**Figure 1:**
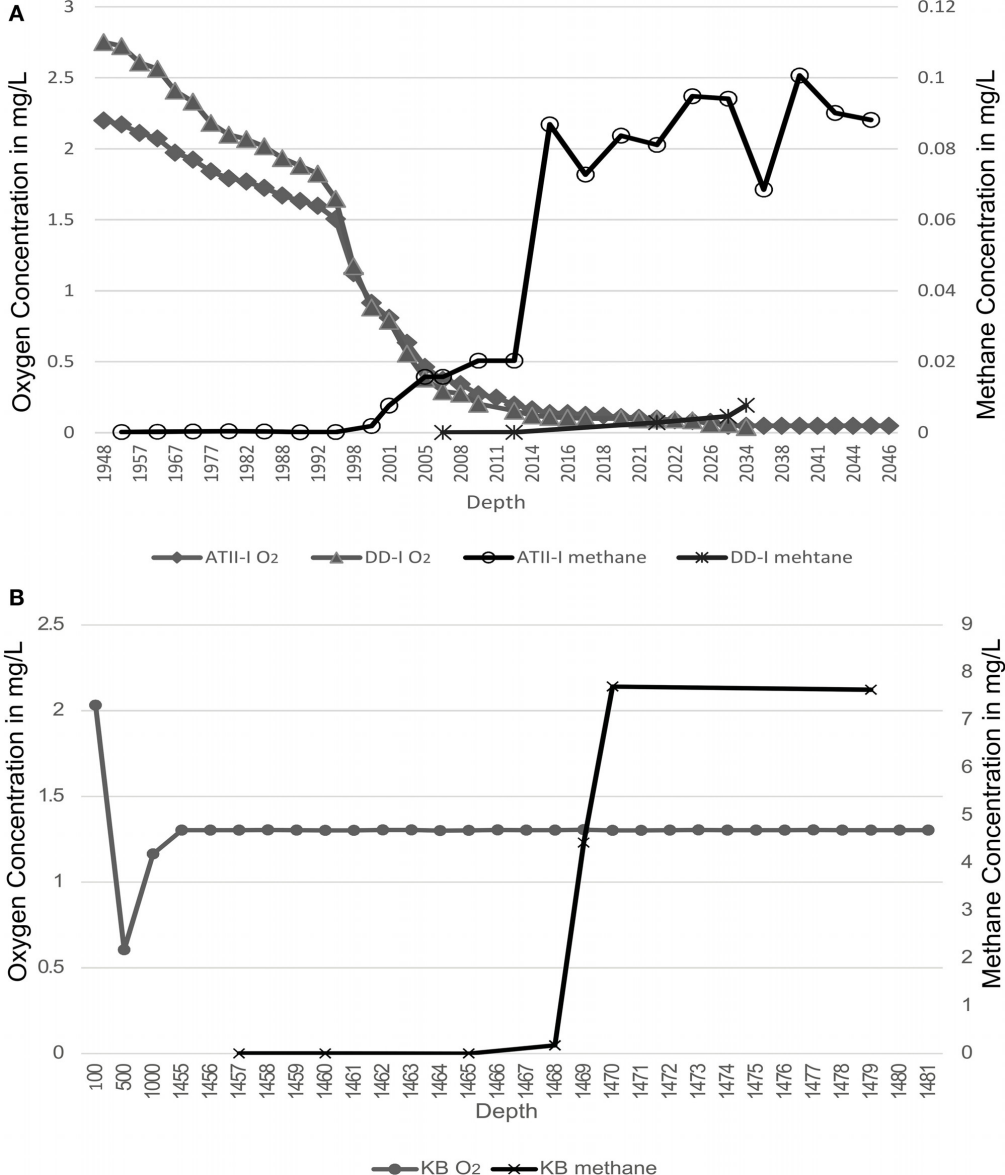
**Methane and oxygen concentrations profiles**. Methane and oxygen concentrations are presented across the brine-seawater layers. **(A)** Atlantis II Deep (ATII-I) and Discovery Deep (DD-I). **(B)** Kebrit Deep (KB). Oxygen profiles are presented on the primary Y-axis, and methane profiles are presented on the secondary Y-axis. The oxygen profiles are based on CTD measurements conducted during sampling in the present study, while the methane profiles are based on measurements from Schmidt et al. (2003).

At the Atlantis II Interface (ATII-I), Discovery Deep Interface (DD-I) and Kebrit Deep Lower Interface (KB-L), 240 liters of water was collected, while 120 liters was collected from the Kebrit Deep Upper Interface (KB-U) during the KAUST Red Sea R/V *Aegaeo* expedition in spring 2010 (Table 1). At ATII, samples were collected from the overlying water column from depths of 50, 200, 700, and 1500 m (Siam et al., 2012; Ferreira et al., 2014). The samples from the ATII overlying water column were used for the subtraction of common reads from the different brine/interface layers. These large volumes of water were collected using shipboard Niskin bottles connected to CTDs (conductivity, temperature, and depth sensors) at the depths indicated in Table 1. Oxygen saturation was measured using a SeaBird DO sensor mounted to the CTDs. For all of the samples, with the exception of KB-U, the CTD was deployed twice at the same depth to collect the required water volume. Sequential microbial size fractionation was performed on Millipore Mixed Cellulose Esters filters (Nitrocellulose/Cellulose Acetate) with pore sizes of 3, 0.8, and 0.1 μm, only the last of which was further processed and analyzed. The filters were collected and stored in sucrose lysis buffer (Rusch et al., 2007) and were held at −20°C until delivery to the laboratory at the American University in Cairo, where they were stored in a −80°C freezer. Oxygen saturation was measured in the three brine-seawater interfaces. However, methane concentrations were obtained from a previous study (Figure 1) (Schmidt et al., 2003).

**Table 1:** **Description of samples**.

**Sample description**	**Latitude/Longitude**	**Sample depth**	**Environmental library description**
Atlantis II interface (ATII-I)	21.60528/38.2025	1996–2025 m	*pmoA*/16S rRNA/Pyrosequencing
Discovery interface (DD-I)	21.28639/38.28722	2026–2042 m	16S rRNA/Pyrosequencing
Kebrit upper interface (KB-U)	24.7187/36.2888	1468 m	*pmoA*/16S rRNA/Pyrosequencing
Kebrit lower interface (KB-L)	24.7187/36.2888	1469 m	16S rRNA/Pyrosequencing

### DNA extraction, pyrosequencing, and construction of functional gene libraries

Total genomic DNA was extracted from each 0.1 μm filter applied to the four aforementioned samples, three separate times for each site. Each filter was cut into small square pieces and was incubated with TE buffer (50 mM EDTA + 10 mM Tris) and 2.5 mg/ml lysozyme at 37°C for 2 h. Subsequently, proteinase K (200 μg/ml) and 1% SDS were added to the filter pieces, followed by incubation overnight at 55°C. The Metagenomic DNA Isolation Kit for Water (EPICENTRE Biotechnologies, Madison, WI, USA) was employed to isolate DNA from each treated filter. DNA was quantified via the Picogreen assay using a NANODROP™ 3300 Fluorospectrometer. The DNA quantities extracted from the ATII-I, DD-I, KB-U, and KB-L samples were 5, 5.5, 9.4, and 6.6 μg, respectively.

Metagenomic libraries for these four different interface layers were constructed as recommended by the GS FLX Roche Titanium library guide, and DNA fragment sizes were selected using the Double SPRI Method (Hawkins et al., 1994). Pyrotag sequence analysis was performed as described in Siam et al. (2012), using universal bacterial primers to amplify the V4–V6 regions of 16S rRNA (Siam et al., 2012). The 16S rRNA pyrosequencing analysis was performed three different times for each sample, and the results were merged. Shotgun sequencing was performed once for each sample. Pyrosequencing of both the 16S rRNA amplicons (486.3 ± 0.04 bp) and metagenomes (327.13 ± 0.06 bp) were performed using 454 GS FLX Titanium technology (454 Life Sciences).

*pmoA* plasmid library construction was performed using the A189f-A682r and the A189f-mb661r primer pairs via the semi-nested approach, as described by Horz et al. (2005). We were only able to amplify the *pmoA* gene (500.4 ± 2.2 bp) from ATII-I and KB-U DNA. Approximately 100 ng of template DNA was used. The first round of PCR was performed in triplicate, and the pooled template was used for the second round of PCR (Horz et al., 2005). The gel-extracted PCR products were cloned into the pGEM®-T Easy Vector (Promega, Madison, WI, USA) according to the manufacturer's instructions and transformed into TOP10 *E. coli*. From the transformed cells, 98 and 96 randomly selected clones from ATII-I and KB-U, respectively, were grown on LB media for DNA extraction. Plasmid DNA extraction was carried out using the R.E.A.L. Prep 96 Plasmid Kit (QIAGEN, Valencia, CA, USA). All of the extracted plasmids were sequenced using a 96-Capillary Sequencer 3730xl DNA Analyzer (Applied Biosystems, Carlsbad, CA, USA). The cloned inserts were amplified using the M13F and M13R sequencing primers (Promega, Madison, WI, USA). Cycle sequencing was conducted in both directions using the BigDye® Terminator v3.1 Kit.

### Bioinformatics analyses

The preprocessing (quality trimming and filtering) of 16S rRNA was performed using the Visualization and Analysis of Microbial Population Structures (VAMPS) website, http://vamps.mbl.edu/ (Sogin et al., 2006; Huse et al., 2014). This procedure was followed by dereplication and chimera checks (both de novo and reference based) using the UCHIME algorithm implemented in USEARCH (Edgar et al., 2011). Rarefaction analysis for 16S rRNA pyrotags was conducted using Quantitative Insights Into Microbial Ecology (QIIME) software, with a 99% similarity threshold (Caporaso et al., 2010). Further alpha diversity analysis measures for the 16S rRNA pyrotags were calculated using QIIME v.1.4. The taxonomic assignments of the 16S rRNA pyrotags were determined using the Global Assignment of Sequence Taxonomy pipeline available through VAMPS (Huse et al., 2008). To reveal the 16S rRNA pyrotags unique to the interfaces, a local BLASTn search with 97% coverage and a 97% identity threshold was performed, aligning the interface layer pyrotags against the ATII water column samples (50, 200, 700, 1500 m). Then, interface pyrotags that showed a hit among the ATII water column samples were eliminated from the VAMPS taxonomic classification. Statistical filtering of the taxa identified from the 16S rRNA pyrotags unique to the interfaces was performed using the two-tailed Fisher's exact test (FET) (Fisher, 1970). Taxa lacking statistically significant differences between samples were eliminated using FET. Then, the pyrotags were normalized based on percentages. FET was carried out using R software (http://www.r-project.org/) with a *p*-value threshold ≤0.05 and Bonferroni correction (R Development Core Team, 2010).

A phylogenetic tree of the 16S rRNA sequences was generated using selected operational taxonomic units (OTUs) from the pyrotags from the ATII-I, KB-U, and KB-L libraries, in addition to selected SILVA nr (release 115) reference sequences (Quast et al., 2013). The high-quality pyrotags were downloaded from the VAMPS web server and clustered using USEARCH with a 0.99 similarity threshold (Edgar, 2010). OTUs and reference sequence pairs were selected from SILVA nr based on annotation by BLASTn, where only hits to methane-oxidizing and methylotrophic species with a percent identity >95% and an *E* < 1e−5 were considered. Multiple sequence alignment was performed with MUSCLE (Edgar, 2004). FastTree was employed to generate the final tree using the GTR substitution model and 100 bootstrap replicates (Price et al., 2009).

Preprocessing of *pmoA* was performed via (1) trimming vector and low-quality bases (sequences with a quality score ≤30 were eliminated) using CodonCode Aligner software (CodonCode Corporation, Dedham, MA, USA), (2) Chimera checks using the UCHIME algorithm implemented in USEARCH (Edgar et al., 2011) (both de novo and with the reference-based approach) and (3) ORF calling using FragGeneScan (Rho et al., 2010). Then, the sequences were confirmed by BLASTx searches against the NCBI nr database dated 12 2013. Of the 98 ATII-I and 96 KB-U sequences only 59 and 80, respectively, were considered good quality DNA sequences and were used for alpha diversity, taxonomic and phylogenetic analyses. The alpha diversity of the *pmoA* libraries was determined using MOTHUR v.1.18.1 (Schloss et al., 2009). A distance threshold of 0.1 (90% nucleotide sequence similarity) was employed (Heyer et al., 2002; Wasmund et al., 2009). The distance matrix was based on multiple sequence alignments generated with the Multiple Sequence Comparison by Log-Expectation (MUSCLE) program (Edgar, 2004). A phylogenetic tree of the pmoA sequences was generated using reference sequences recruited by a local BLASTx search (maximum E-value of 1e−5 and 100 hits/sequence) was carried out on the *pmoA* clonal inserts against the GenBank non-redundant database (December 2013) to identify the most closely related cultured and uncultured methanotrophs for each sequence; other reference sequences of cultured methanotrophs and *pxmA* genes were also included (Tavormina et al., 2011). The *pmoA* DNA sequences from ATII-I and KB-U were first clustered into OTUs with a distance threshold of 0.1 using USEARCH (Schloss et al., 2009). The deduced protein sequences of the *pmoA* OTUs and selected references were employed in the construction of the phylogenetic tree. Multiple sequence alignment was performed using MUSCLE (Edgar, 2004). A maximum likelihood phylogenetic tree was generated using PhyML, with 5 random seeds AND confidence values calculated using 100 bootstrap replicates (Stamatakis, 2006; Guindon et al., 2010). The LG substitution model and a gamma distribution were utilized. FigTree (http://tree.bio.ed.ac.uk/software/figtree/), and Archaeopteryx was employed for tree editing (Han and Zmasek, 2009).

Metagenomic reads were deposited in the Meta-Genome Rapid Annotation using Subsystem Technology MG-RAST-CLOUD (version 3) server (Meyer et al., 2008). A protein-based phylogenetic analysis was performed through MG-RAST server sequence similarity searches against the M5NR database, with a maximum *E*-value of 1e−5, a minimum identity of 50% and a minimum alignment length of 20 aa (Meyer et al., 2008). Similar to the pyrotag analyses, statistical filtering of the metagenomic reads was performed using FET, and the reads were then normalized based on percentages.

Recruitment to Kyoto Encyclopedia of Genes and Genomes (KEGG) categories was performed using BLASTx, employing the reads from each of the brine sequence sets as queries against a modified KEGG database using an *E*-value of 1e−5. A non-metric multidimensional scaling (NMDS) analysis of the reads recruited to the methane metabolism KEGG pathway was performed using the R package vegan (R Development Core Team, 2010; Oksanen et al., 2013). Standardization and dissimilarity measures were applied as previously described in Anderson et al. (2006). The matrix was normalized to the total count of recruited reads, scaled and log transformed to base 2, and the modified Gower index was employed as a dissimilarity measure (Anderson et al., 2006). An ordination in 2 NMDS dimensions and 3D space was performed using the NMDS function in the vegan package (Oksanen et al., 2013). KEGG orthologous groups were clustered using hierarchical clustering (Suzuki and Shimodaira, 2006). The classification of KEGG orthologous groups into clusters represented in the NMDS was performed based on the modified Gower distance matrix, the complete linkage method and 1000 bootstrap replicates using the pvclust package (Suzuki and Shimodaira, 2006). The cluster solution was selected using the silhouette criterion (Kaufman and Rousseeuw, 1990; Maechler et al., 2013). A heat map representation was generated using the gplots package (Warnes et al., 2011) via hierarchical clustering with a Spearman correlation distance matrix, and the complete linkage clustering method was applied to normalized log-transformed values.

The obtained *pmoA* sequences were deposited in GenBank under accession numbers KJ175561–KJ175699. The direct DNA shotgun pyrosequences and 16S rRNA pyrotags were deposited in the Short Read Archive (SRA) database, listed under BioProject PRJNA219363.

## Results

### 16S rRNA, pmoA and shotgun metagenomic libraries generated from red sea brine-seawater interfaces

More than 60500 bacterial 16S rRNA pyrotags were generated from ATII-I, DD-I, KB-U and KB-L. Taxonomic assignment of OTUs to major bacterial groups led to the identification of 97, 81, 74, and 58% of the bacterial OTUs in ATII-I, DD-I, KB-L, and KB-U, respectively (Table 2). The greatest number of OTUs not assigned to known bacterial taxa was observed in the KB-U library (Table 2). The difference between the total number of pyrotags and bacterial reads (Table 2) represents non-specific tags that are taxonomically assigned to an archaeal/eukaryal origin. The gene encoding the structural polypeptide pMMO (*pmoA*) was only amplified from the ATII-I and KB-U samples. Following the elimination of low-quality reads, a total of 59 and 80 sequences were recovered from the ATII-I and KB-U libraries, respectively. Additionally, ~4 million metagenomic (454) reads were generated from the four different Red Sea brine-seawater interface layers. The percentage of metagenomic (454) reads that were phylogenetically assigned to a known bacterial origin based on protein sequences varied in the different samples tested; the highest number of bacterial reads was identified in KB-U (~50%) and the lowest in DD-I (~17%). The greatest numbers of metagenomic (454) reads without a match to a public database (~75%) were observed in the ATII-I and DD-I layers (data not shown).

**Table 2:** **Description of the different libraries generated in this study**.

**Samples**	**Samples**	**16S rDNA Reads #/%**	***pmoA* Reads #/%**	454^**^ Reads #/%
Bacteria	AT II-I	16104/96.9	59/100	119158/19.7
	DD-I	13376/81.3	NA	115943/17.6
	KB-U	8069/58	80/100	707139/49.9
	KB-L	9942/73.5	NA	451616/32.9
Total	AT II-I	16618	59	604000
	DD-I	16457	NA	658619
	KB-U	13904	80	1417636
	KB-L	13523	NA	1370213
Unknown^*^	AT II-I	4/0.02	NA	23299/3.9
	DD-I	755/4.6	NA	27301/4.1
	KB-U	689/4.9	NA	120459/8.5
	KB-L	501/3.7	NA	79137/5.8

* *Unknown represents the number of reads not assigned to any known Taxa*.

*NA, Not Available*.

** *The table presents only reads assigned to a bacterial origin (including unknown). The 454 archaeal, eukaryotic and reads showing no hits to public databases are not presented and represent the remaining percentage of reads*.

### Bacterial communities in the brine-seawater interface layers

**Figure 2:**
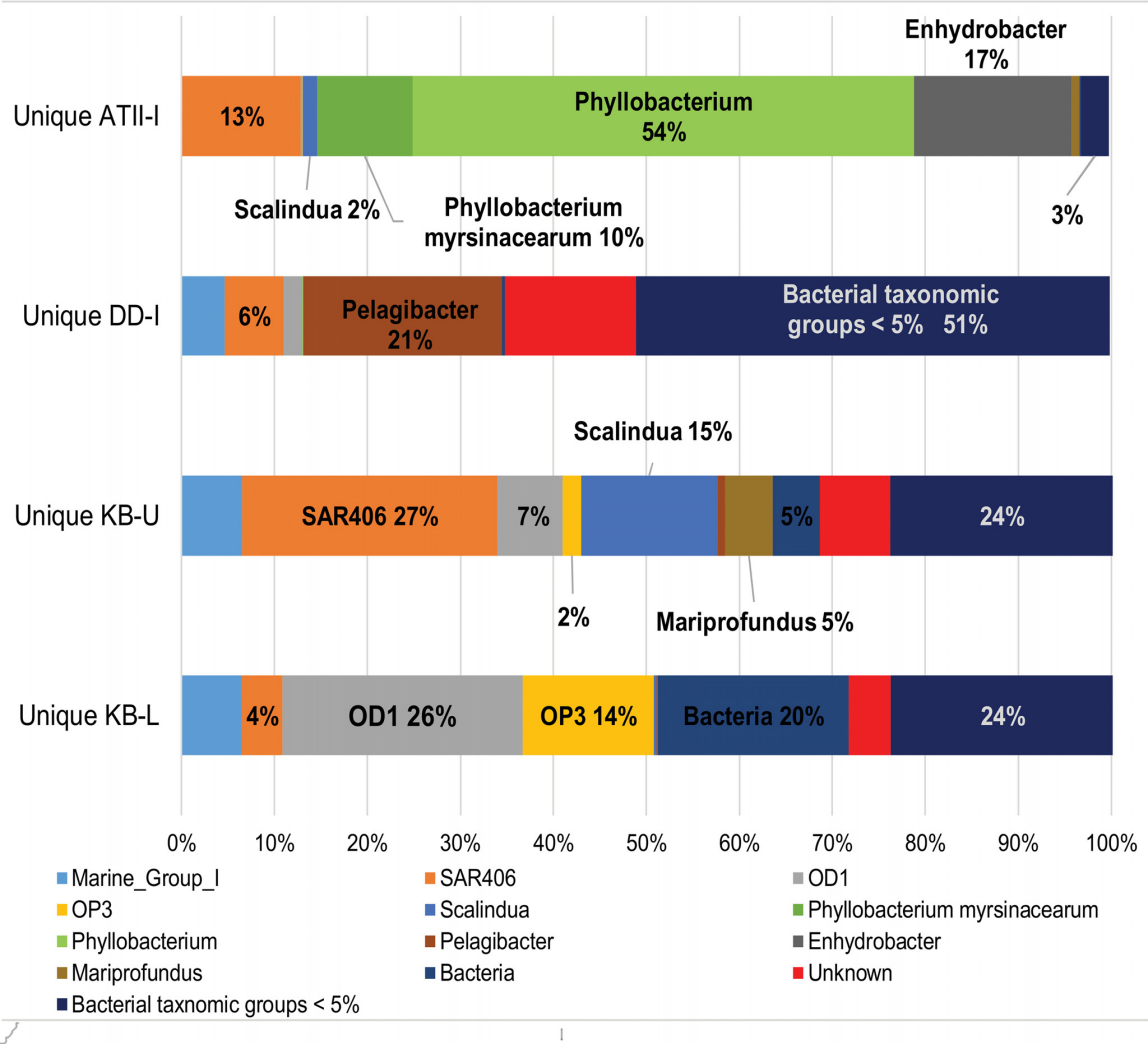
**Assignment of unique 16S rRNA sequences from the brine-seawater interface to major bacterial taxonomic groups**. Representation of the significant unique 16S rRNA OTUs obtained at the interfaces assigned to major bacterial taxonomic groups in the four brine-seawater interface layers. The graph is based on VAMPS taxonomic classification of statistically significant reads after subtracting 16S rRNA sequences shared with the ATII water column samples. Bacteria = pyrotags assigned to bacterial origin, Unknown = pyrotags assigned to an unknown origin, Bacterial taxonomic groups <5% = other bacterial taxonomic groups representing less than 5% of the pyrotag library in any of the four samples. Marine_Group_I=non-specific amplification from archaeal sequences.

Taxonomic assignment of OTUs to the major bacterial groups was performed for the 16 s rRNA pyrotag libraries. We eliminated 16 s rRNA pyrotags found in the Atlantis II overlying water column by subtracting similar reads from the brine-seawater interface pyrotags (described in the Materials and Methods). Thus, we excluded pyrotags that were potentially shared with the overlying water column. It has been shown that the overlying water columns at ATII and DD are relatively homogenous at similar depths (Qian et al., 2011), and we therefore utilized data from the ATII overlying water column in our analysis. The number of unique brine-seawater interface pyrotags were 13357, 3407, 8234, and 10981 for ATII-I, DD-I, KB-U, and KB-L, respectively (Supplementary Table 1). Alpha diversity analyses of the 16S rRNA pyrotags (from the entire bacterial community) indicated that the KB interface layers (both lower and upper) showed the highest diversity, followed by DD-I and, finally, ATII-I (Supplementary Table 2). In the following order, the ATII-I unique OTUs were dominated by unknown phylotypes of *Phyllobacterium* (Phyllobacteriaceae) and *Enhydrobacter* (Moraxellaceae), unknown genera of SAR406 and *Phyllobacterium myrsinacearum* (order: Rhizobiales) (Figure 2, Supplementary Table 3), while unknown phylotypes of *Leucothrix* (order: Thiotrichales), *Scalindua* (order: Planctomycetales), and *Mariprofundus* (order: Mariprofundales) and an unknown class of OD1 represented less abundant and unique ATII-I OTUs (Supplementary Table 3). Unknown classes of OD1 and OP3 dominated the unique KB-L OTUs (Figure 2, Supplementary Table 3). The less abundant and unique KB-L OTUs included unknown genera of Desulfobacteraceae (order: Desulfobacterales), an unknown phylotype of *Sulfurovum* (order: Campylobacterales), and unknown classes of WS3, OP1, and TM6, along with an unknown order of Spirochaetes (Supplementary Table 3). The unique KB-U OTUs were dominated by unknown genera of SAR406, unknown phylotypes of *Scalindua*, *Nitrospira* (order: Nitrospirales), and *Methylobacter lutues* (order: Methylococcales), and an unknown class of OD1 (Figure 2, Supplementary Table 3). The less abundant and unique KB-U OTUs were *Methylophaga aminisulfidivorans* (order: Thiotrichales), unknown phylotypes of *Methylobacter* and *Iamia* (order: Acidimicrobiales), unknown genera of Rhodospirillaceae (order: Rhodospirillales) and an unknown family of SAR324 (Supplementary Table 3). An unknown phylotype of *Pelagibacter* (order: Rickettsiales) dominated the DD-I unique OTUs (Figure 2, Supplementary Table 3), while the less abundant and unique OTUs found in DD-I consisted of unknown phylotypes of *Myxococcus* (order: Myxococcales) (Supplementary Table 3).

### Aerobic methanotrophic communities at the brine-seawater interface identified based on 16S rRNA pyrotags and shotgun pyrosequencing metagenomic analyses

**Figure 3:**
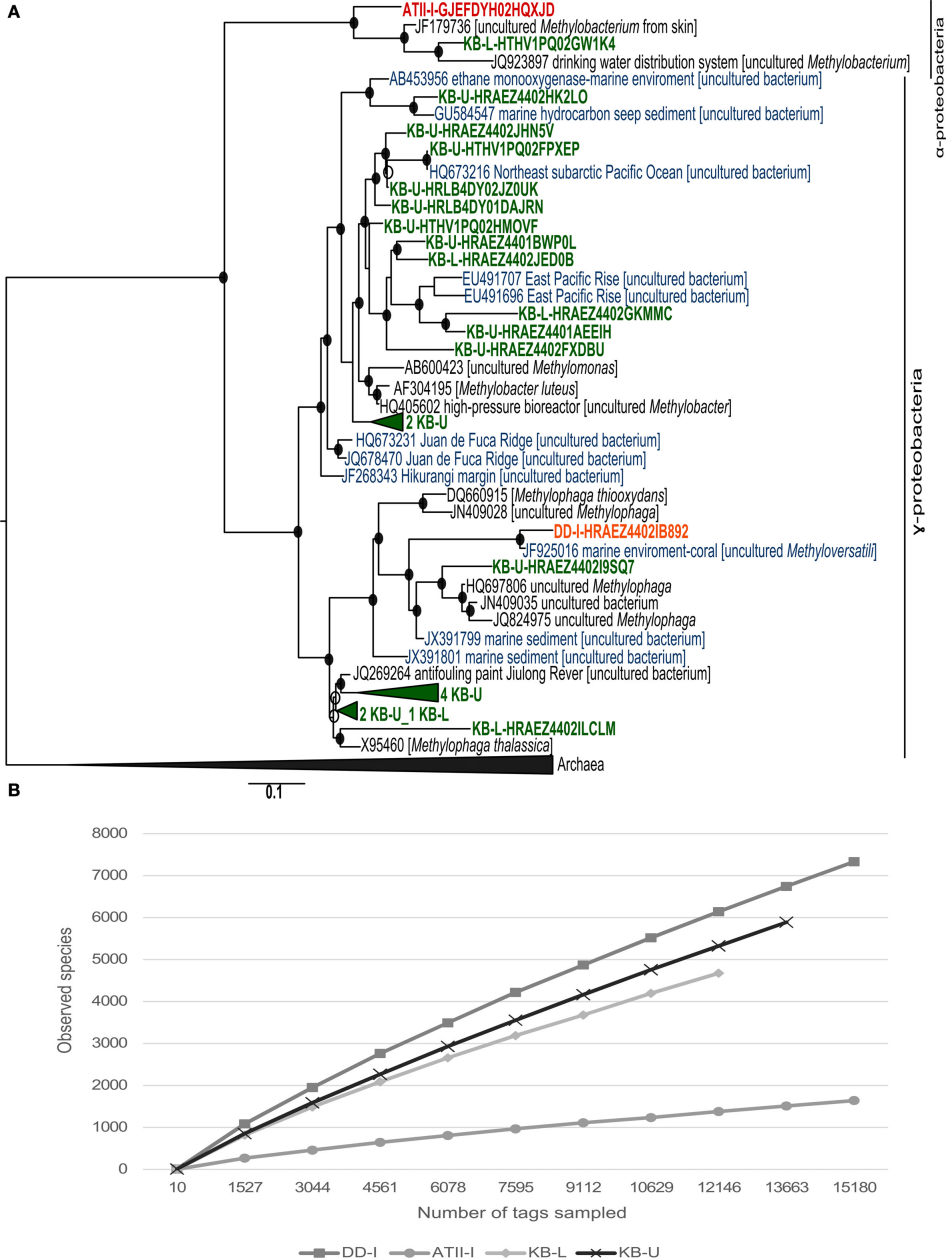
**16S rRNA phylogenetic tree and species richness of aerobic methanotrophs. (A)** Nucleotide phylogeny of the predicted 16S rRNA pyrotags generated from ATII-I (red), DD-I (orange) and KB-U/KB-L (green). Blue sequences represent 16S rRNA derived from marine environments. Red Sea brine pool OTUs based on 99% sequence identity were compared with SILVA 16S rRNA. The tree was generated using an 1891-bp alignment. The numbers of sequences collapsed in the selected node are indicated adjacent to the sample name. The trees were calculated using the maximum-likelihood approach with 100 bootstrap resampling. Bootstrap values ≥50% are depicted as open circles and those ≥70% with closed circles. The scale bar represents 10% estimated sequence divergence. **(B)** Species richness in the ATII-I, DD-I, KB-U and KB-L brine-seawater interface layers based on 16S rRNA pyrotag rarefaction curve analysis. Phylotypes were generated based on a distance threshold of 0.01.

The VAMPS taxonomic classification (Table 3 and Supplemental Table 3) and the phylogenetic clustering of 16S rRNA sequences against methanotroph and methylotrophic 16S rRNA sequences retrieved from the SILVA nr database (Figure 3A) were used to assess the phylogeny of the known aerobic methanotrophs inhabiting the brine-seawater interface layers (Hanson and Hanson, 1996; Dunfield et al., 2007; Pol et al., 2007; Islam et al., 2008; Murrell, 2010; Qian et al., 2011; Siam et al., 2012). The results of the rarefaction analysis of the 16S rRNA showed that only ATII-I has reached saturation (Figure 3B).

**Table 3:** **Reads assigned to bacterial genera involved in aerobic methane oxidation across the different brine-seawater interface layers**.

**Genus**	**ATII-I**			**KB-U**			**KB-L**		**DD-I**		**Water column**	
	**16S read _#/%_**	***pmoA* read _#/%_**	454^**^ read _#/%_	**16S read _#/%_**	***pmoA* read _#/%_**	454^**^ read _#/%_	**16S read _#/%_**	454^**^ read _#/%_	**16S read _#/%_**	454^**^ read _#/%_	**16S read _#/%_**	454^**^ read _#/%_
*Methylomarinum*	–	7/11.9	–	–	2/2.5	–	–	–	–	–	–	–
*Methylobacter*	–	–	32/0.02	239/2.9	78/97.5	2634/0.34	11/0.1	243/0.05	–	185/0.11	–	1551/0.18
*Methylococcus*	–	48/81.4	72/0.05	–	–	2835/0.3	–	692/0.14	–	347/0.20	–	5057/0.39
*Methylomicrobium*	–	–	–	1^*^	–	6^*^	–	4^*^	–	–	–	4^*^
*Methylomonas*	–	–	1^*^	1^*^	–	19^*^	–	4^*^	–	1^*^	–	4^*^
*Methylocaldum*	–	–	–	–	–	2^*^	–	–	–	–	–	–
*Methylocystis*	–	–	105/0.07	–	–	185/0.02	–	44/0.01	–	49/0.03	–	501/0.07
*Methylosinus*	–	–	71/0.05	–	–	366/0.05	–	92/0.02	–	60/0.03	–	1342/0.16
*Methylocella*	–	–	186/0.13	–	–	484/0.06	–	192/0.04	–	131/0.08	–	3615/0.39
*Methylacidiphilum*	–	–	21/0.012	–	–	764/0.1	–	725/0.14	–	137/0.18	–	1329/0.16
*Methylothermus*	–	–	–	–	–	1^*^	–	–	–	–	–	–
*Methylocapsa*	–	–	2^*^	–	–	–	–	–	–	–	–	9^*^
*Methylosarcina*	–	–	–	–	–	–	–	–	–	–	–	1^*^
*Uncultured methanotrophs*	1^*^	4/6.7	–	10^*^	–	4^*^	1^*^	–	2^*^	–	8^*^	–

* *Not statistically significant*.

** *454 percentages are based on MGRAST recruitment to the total identified reads*.

VAMPS taxonomic classification identified 250 statistically significant bacterial 16S rRNA pyrotags assigned to the Methylococcaceae family in the Kebrit interface layers (Table 3). Among the 16S rRNA tags, ~2.9% (239 reads) and 0.1% (11 reads) of the 16S rRNA tags from KB-U and KB-L, respectively, were taxonomically assigned to the genus *Methylobacter* (Table 3). The ATII-I and DD-I OTUs represented 8% (4% each) of the 25 total methylotrophic Red Sea interface OTUs (Figure 3A), while the KB-L and KB-U OTUs represented 20 and 72% of the total number, respectively (Figure 3A).

Phylogenetically, 18 OTUs from KB-U were related to two major groups: type I methanotrophs and methylotrophic bacteria (Figure 3A). Seven of the KB-U OTUs were found to be related to the methylotrophic bacteria of the *Methylophaga* genus. The greatest number of KB-U OTUs (9) were phylogenetically related to several marine methanotrophs, with the genus *Methylobacter* being the most closely related cultured methanotroph (Figure 3A). Moreover, two OTUs represented an out-group of the *Methylobacter* clade.

The KB-L interface possessed five OTUs that were phylogenetically related to methanotrophs and methylotrophs. Two OTUs were related to the methylotrophic bacteria of the *Methylophaga* genus, while two were related to several marine methanotrophs, with the genus *Methylobacter* representing the most closely related cultured group of methanotrophs (Figure 3A). One OTU was clustered with 16S rRNA sequences from the methylotroph *Methylobacterium* (α-proteobacteria).

For ATII-I, only one 16S rRNA OTU was phylogenetically assigned to *Methylobacterium*, while DD-I presented only one 16S rRNA OTU that was phylogenetically related to *Methylophaga*.

Reads assigned to aerobic methanotrophs were identified in the four libraries using sequence similarity searches of ~4 million metagenomic (454) reads. Similar to the 16S rRNA analyses, the pyrosequencing read rarefaction analyses showed that only ATII-I has reached saturation (data not shown). The percentages of reads corresponding to aerobic methanotrophs in the different libraries were quite low (~0.5%), though the highest percentage assigned to the *Methylobacter* genus was found in KB-U (~0.3%) (Table 3). Reads assigned to type II methanotrophs were only present in the four shotgun pyrosequencing libraries.

### Aerobic methanotrophic communities at the brine-seawater interface based on /*pmoA* phylogenetic analyses

**Figure 4:**
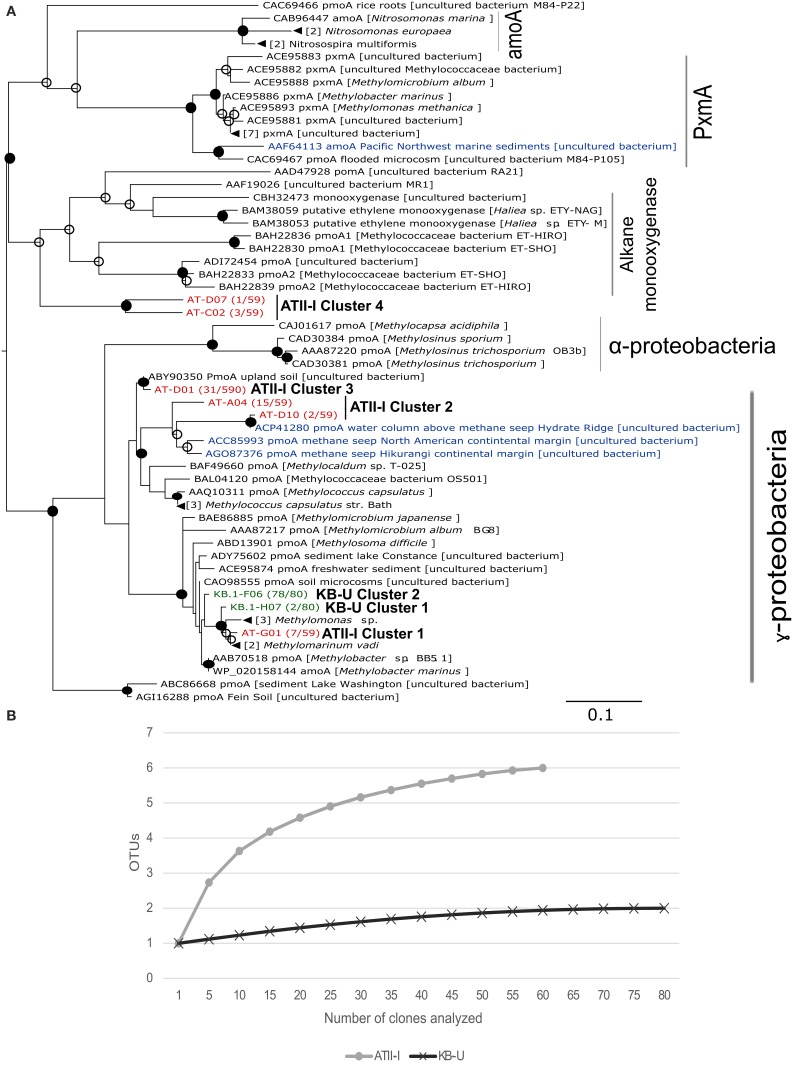
***pmoA* phylogenetic tree and species richness of aerobic methanotrophs. (A)** Deduced amino acid phylogeny of predicted *pmoA* sequences amplified from ATII-I (6 OTUs-red) and KB-U (2 OTUs-green) based on 90% nucleotide sequence identity. The Red Sea brine pool OTUs were compared with deduced amino acid sequences for *pmoA* derived from other marine environments (blue), soil and cultured methanotrophs. The number between parentheses, following the sequence name, represents the number of sequences within the library that belong to the represented OTUs. The tree was generated using 168 aa. The numbers of sequences collapsed in the selected node are indicated adjacent to the sample name. The tree was calculated using the maximum-likelihood approach with 100 bootstrap replicates. Percentages greater than 50% of bootstrap resampling are indicated with an open circle and those greater than 70% with a closed circle near the nodes. The scale bar represents 10% estimated sequence divergence. **(B)** Species richness in the Atlantis II Deep and Kebrit Deep brine-seawater interphase layers based on *pmoA* clone library rarefaction curve analysis. Phylotypes were generated based on a distance threshold of 0.1.

We further investigated the aerobic methanotrophic communities using clone libraries of the particulate methane monooxygenase gene (*pmoA*). We were only successful in amplifying *pmoA* from ATII-I and KB-U. A total of 139 clones were sequenced for the two samples studied, with 59 clones from ATII-I and 80 from KB-U being identified as *pmoA*. Rarefaction analysis of the *pmoA* clone libraries indicated that sufficient numbers of *pmoA* genes were amplified from the aerobic methanotrophic community (Figure 4B). In contrast to the alpha-diversity 16S rRNA analyses, the *pmoA* aerobic methanotrophic communities showed more diversity in ATII-I than KB-U (Supplementary Table 2). Six *pmoA* OTUs were identified in ATII-I, while only two were identified in KB-U (Table 3). The sequences were aligned to the GenBank database, and similarity searches between the sequences were conducted using BLASTx. A phylogenetic tree was generated using the maximum likelihood method. Both ATII-I and KB-U clones were phylogenetically classified as type I methanotrophs γ–proteobacteria).

The obtained *pmoA* sequences were divided into six distinct clusters; four were unique to ATII-I, and two were unique to KB-U. The clusters are presented and numbered in descending order in the tree (Figure 4A). In the ATII-I *pmoA* library, the majority (~81%) (ATII-I cluster 3 and 4) of the *pmoA* clones appeared to be related to different uncultured methanotrophs (Figure 4A, Table 3 and Supplementary Table 4), with the genus *Methylococcus* (Table 3) and the species *Methylococcus capsulatus* (Supplementary Table 4) representing the most closely related cultured methanotrophs. Of the ATII-I *pmoA* clones (ATII-I cluster 1), 12% were closely related to the newly discovered aerobic methanotroph *Methylomarinum vadi*, while 7% (ATII-I cluster 4) were not assigned to any cultured methanotrophs (Figure 4A, Table 3 and Supplementary Table 4). Phylogenetically, two OTUs (ATII-I cluster 2) of the six clustered with uncultured marine methanotrophs identified from water and sediment samples from methane seeps. This cluster showed the *Methylococcus* genus as the most closely related group of cultured methanotrophs (Figure 4A). One OTU (ATII-I cluster 3) represented an out-group of the marine methanotroph clade and was related to an uncultured bacterium from landfill soil (Figure 4A). Seven reads (one OTU-ATII-I cluster 1) from the ATII-I library were phylogenetically closely related to the newly discovered genus *Methylomarinum* (species *Methylomarinum vadi*) (Hirayama et al., 2013). Interestingly, of the 59 clones that were related to several uncultured and cultured methanotrophs, our sequence similarity searches showed 4 highly divergent clones (two OTUs-ATII-I cluster 4) presenting less than 70% identity to any known *pmoA* sequence (Figure 4A and Supplementary Table 4). These OTUs were represented in the tree as a divergent clade of alkane monooxygenases. The alkane monooxygenases and partial *pxmA* genes were shown in the tree as an out-group of both Type I and Type II methanotrophs (Figure 4A).

In the KB-U library, 78 clones/one OTU-KB-U cluster 2 (97.5%) were closely related to the genus *Methylobacter* according to sequence similarity searches, while phylogenetically, they showed an unclear affiliation with the *Methylobacter* clade. The other KB-U OTU (KB-U cluster 1) represents an out-group of the *Methylomarinum*/*Methylomonas* clade (Figure 4A).

### Methane metabolic profiles in the brine-seawater interface layers based on shotgun pyrosequencing metagenomic reads

**Figure 5:**
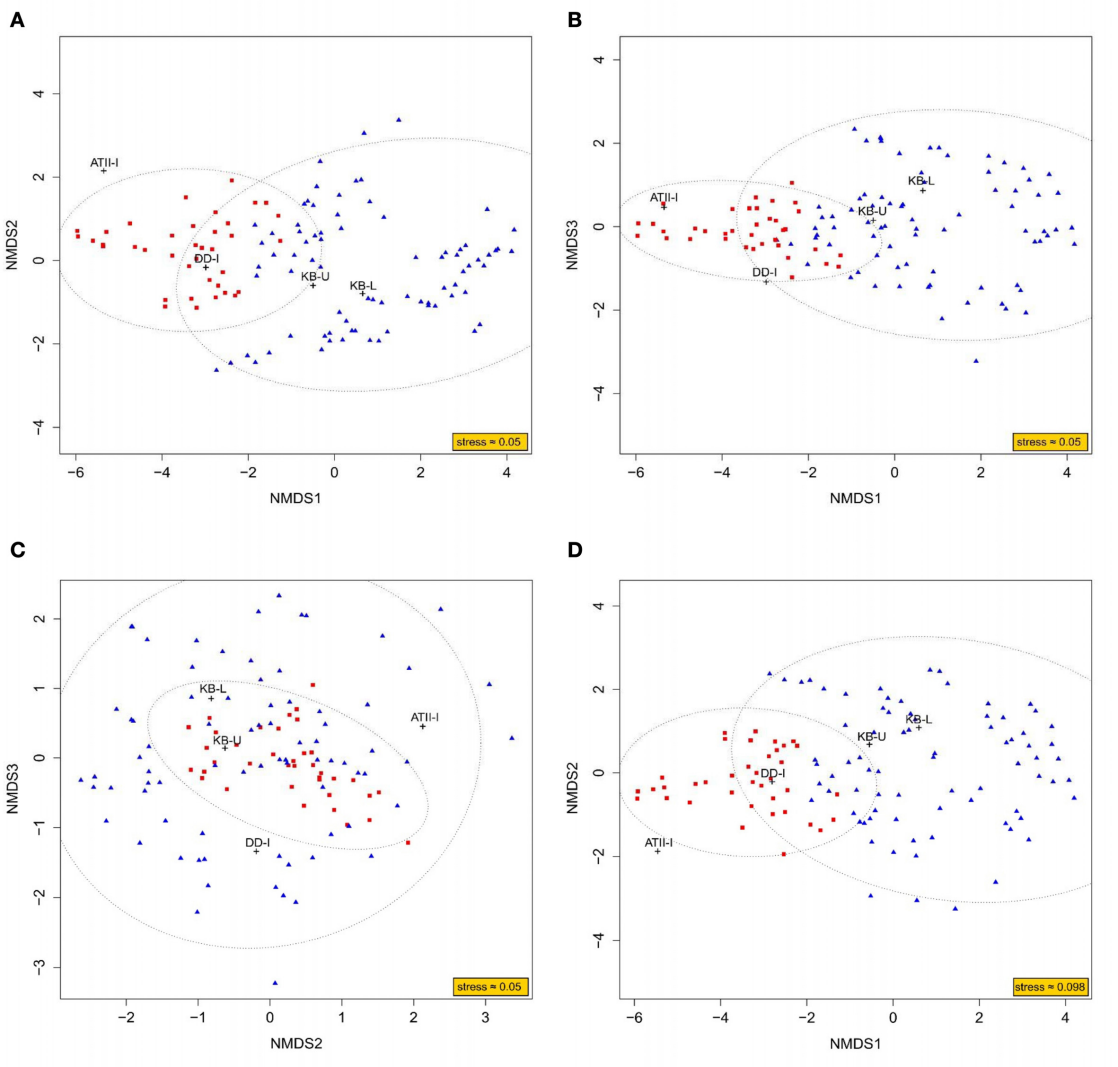
**NMDS ordination plot based on KEGG mapping to methane metabolism pathways**. Representation of the NMDS analysis in 3D **(A–C)** and 2D **(D)**. The analysis considers each dataset of reads obtained through Red Sea interface shotgun pyrosequencing as a variable. The read counts recruited to methane metabolism-related KEGG orthologous groups were considered as an observation. The ordination of observations is represented by either blue triangles (cluster 1) or red squares (cluster 2). While the black crosses represent the ordination of variables, which is deduced from the weighted average of their respective observations. An ellipse around the centroid of each cluster depicts 95% confidence in the assignment of observations to the cluster.

To obtain a better understanding of the methane metabolism occurring in the three brine-seawater interfaces, metagenomic (454) reads were mined for enzymes involved in the methane pathway within the KEGG database. We present an NMDS analysis of putative enzymes involved in methane metabolism in the interface brine samples in both 3D and 2D (Figure 5). The NMDS analysis presented in 3D converged with a stress value of ≈0.054, whereas the 2D presentation converged with a stress value of ≈0.098. The ordination of KEGG orthologous groups in the NMDS showed two different clusters. Cluster 1 included KB-U and KB-L samples, with 95% confidence. ATII-I and DD-I did not group together or with the KB samples. However, ATII-I was markedly dissimilar from the other analyzed samples. Of 133 enzymes involved in methane metabolism identified in our samples, only 37 were associated with aerobic methane oxidation. Those 37 enzymes included major enzymes involved in methane oxidation and formaldehyde assimilation in aerobic methanotrophs. The key enzyme methane monooxygenase was represented by 5 KOs (KEGG Orthologs): K10944 (pMMO subunit A), K10945 (pMMO subunit B), K10946 (pMMO subunit C), and K16158 (sMMO subunit A), which were mainly present in KB-U, while K16161 (sMMO subunit C) was present in all layers, showing higher abundance in KB-U (Table 4, Supplementary Table 5). The other identified enzymes involved in the oxidation of methane to carbon dioxide were formaldehyde dehydrogenase and formate dehydrogenase, which were detected in DD-I and all layers, respectively, while methanol/alcohol dehydrogenase was absent. Formaldehyde dehydrogenase (K00148) was only found in DD-I. Formate dehydrogenase (K00122, K00123, K00124, K00125, K00127, K05299, K08348, and K15022) was mainly detected in Kebrit layers and was found at a low abundance in DD-I and ATI-II (Supplementary Figure 1 and Supplementary Table 5). All of the known enzymes involved in methane oxidation via the tetrahydromethanopterin pathway were detected in both Kebrit layers and ATII-I, but at a lower abundance. In DD-I, the enzyme methylenetetrahydromethanopterin dehydrogenase was not detected (Supplementary Figure 1 and Supplementary Table 5).

**Table 4:** **Comparison of the sMMO and pMMO reads obtained from the brine-seawater interfaces**.

**Sample description**	**Sample depth**	**Oxygen concentration**	Methane concentration^*^	**sMMO 454 reads**	**pMMO 454 reads**	***pmoA* clones**
DD-I	2026–2042 m	0.06–0.04 mg/L	0.005–0.008 mg/L	9	0	0
ATII-I	1996–2025 m	1.5–0.8 mg/L	0.0002–0.09 mg/L	8	0	59
KB-L	1469 m	1.31 mg/L	4.43 mg/L	10	0	0
KB-U	1468 m	1.30 mg/L	0.17 mg/L	30	9	80

* *Methane concentration is based on Schmidt et al. (2003)*.

Major enzymes known to be involved in formaldehyde assimilation through the serine pathway were identified mainly in KB-U and at a lower abundance in KB-L, DD-I, and ATII-I, with the exception of glycerate kinase. Only two enzymes involved in the other formaldehyde assimilation pathway (the ribulose monophosphate cycle) were identified, mainly in the Kebrit Interface: 3-hexulose-6-phosphate synthase (K08093) and 6-phospho-3-hexuloisomerase (K08094 and K13831) (Supplementary Figure 1 and Supplementary Table 5).

## Discussion

This study is the first to investigate and compare the aerobic methane-oxidizing communities present at three Red Sea brine-seawater interfaces, at the Atlantis II, Discovery and Kebrit Deeps. Serial water filtration of the brine-seawater interface samples allowed us to focus on free-living prokaryotes. The density gradient created at the brine-seawater interface causes organic and inorganic matter from the overlying seawater to be trapped (Hartmann et al., 1998; Schmidt et al., 2003). Therefore, the microbial communities attached to these particles as well as those living in symbiosis with eukaryotic microorganisms are likely to have been excluded from our study due to the applied serial filtration method. Samples of 120 liters collected twice from all sites, with the exception of KB-U, were filtered through separate filters. The different free bacterial communities involved in methane oxidation were assessed using three different approaches: metagenomic (454) libraries, 16S rRNA pyrotags and phylogenetic analysis of *pmoA* sequences.

The four different brine-seawater interface layers exhibited diverse bacterial communities. Moreover, each brine-seawater interface exhibited unique dominant taxa. ATII-I was dominated by the genera *Phyllobacterium* (order: Rhizobiales) and *Enhydrobacter* (order: Pseudomonadales). *Phyllobacterium* species are known to be involved in aromatic hydrocarbon degradation and have been identified in different marine ecosystems (von der Weid et al., 2008; Shao et al., 2010). This might explain the dominance of *Phyllobacterium* in ATII-I, due to being a hydrocarbon-rich layer (Burke et al., 1981; Faber et al., 1998; Schmidt et al., 2003). The Kebrit brine-seawater interface layers (KB-U and KB-L) were mainly dominated by the marine Candidatus taxa OP3 and SAR406 (Gordon and Giovannoni, 1996; Glockner et al., 2010; Kumar and Saravanan, 2010). The Candidatus OD1 phylum (dominant in KB-L) is known to occur in marine environments characterized by notable concentrations of sulfur compounds (Harris et al., 2004). The dominance of the marine ammonium oxidizer *Scalindua* in the KB-U layer can be explained by the presence of a high hydrogen sulfide concentration favoring the sulfur reduction of *Planctomycetes* (Elshahed et al., 2007). The most dominant bacteria in DD-I belonged the ubiquitous marine bacterial genus *Pelagibacter* (Morris et al., 2002; Rappe et al., 2002).

The Atlantis II Deep is considered to be the largest, hottest, and saltiest pool in the Red Sea and is characterized by particularly high concentrations of heavy metals relative to other brine pools (Antunes et al., 2011b). Studies examining hydrocarbon concentrations and stable carbon isotopes have provided evidence of methane oxidation at the so-called “anoxic (brine)-oxic (seawater) boundary” (Schmidt et al., 2003), or the interface (Faber et al., 1998; Hartmann et al., 1998; Schmidt et al., 2003). The present study relies on a previously recorded methane stable isotope ratio obtained in 1997 (Schmidt et al., 2003), which is a limitation of our study, as we cannot correlate the abundance and diversity of aerobic methanotrophs with current methane levels. Potential methane-producing Archaea have been identified in the brine pool (Bougouffa et al., 2013) and in different sediment subsections of the Atlantis II Deep (Siam et al., 2012). However, aerobic methanotrophs were not previously identified at the ATII-I interface (Bougouffa et al., 2013). The present work revealed only one 16S rRNA pyrotag that was taxonomically assigned to uncultured aerobic methane-oxidizing bacteria in ATII-I (Table 3). The detection of a small number (or absence) of OTUs assigned to aerobic methanotrophs based on 16S rRNA gene sequences was previously reported in sediment samples from the Timor Sea methane seeps in Australia and in samples from two seep systems along the North American margin in California (Tavormina et al., 2008; Wasmund et al., 2009). It is unclear why the 16S rRNA recovered from these methane seeps did not indicate the presence of the aerobic methanotrophs, whose sequences were amplified using *pmoA* primers (Tavormina et al., 2008, 2010). This disparity could be due to the low abundance of aerobic methanotrophs compared with the total microbial community. Alternatively, the 16S rRNA universal primers may show specificity limitations, masking the amplification of sequences from aerobic methanotrophs. This situation imposes a limitation on the 16S rRNA approach regarding the amplification and detection of sequences from aerobic methanotrophs.

Similar to these studies, the *pmoA* gene phylogenetic analysis provided a comprehensive understanding of the aerobic methanotrophic communities oxidizing methane seeping from this pool. The majority (~88%) of *pmoA* sequences from ATII-I were divergent from any known cultivated aerobic methanotroph (Supplementary Table 4). Furthermore, ~32% of the *pmoA* sequences obtained in this study show <90% identity with sequences in the GenBank database, suggesting that more than 25% of the aerobic methanotrophs in the ATII-I layer exhibit sequences that are largely dissimilar from all of the sequences that have been reported previously in culture-dependent or independent studies. The OTUs retrieved from ATII-I were clustered into four clusters within the deduced amino acid phylogenetic tree (Figure 4A). The majority of the *pmoA* clones in the ATII-I library (ATII-I cluster 3) were closely related to an uncultured bacterium identified from alkaline landfill soil (Chang et al., 2010), while ~28% (ATII-I cluster 2) were closely related to a novel lineage of marine-specific aerobic methanotrophs identified at different sites, including along the North American margin and Deep-Sea Methane Seeps at the Hikurangi continental margin (Tavormina et al., 2008; Ruff et al., 2013). ATII-I cluster 1 was closely related to the newly discovered bacterium *Methylomarinum vadi* (Hirayama et al., 2013). Interestingly, the ATII-I cluster 4 sequences shared less than 70% identity with the best GenBank hit (Supplementary Table 4) and were phylogenetically presented as an out-group of alkane monooxygenases. The similarity of this cluster to ethylene-assimilating marine bacteria (Supplementary Table 4) Suzuki et al. (2012) suggested that these sequences belong to a unique pMMO lineage that is present in ATII-I and possibly other similar environments.

Although the Discovery Deep is also characterized by relatively high concentrations of hydrocarbons, including methane seeping from the brine to the overlaying brine-seawater interface (Faber et al., 1998; Hartmann et al., 1998; Schmidt et al., 2003), the concentration of methane in the Discovery Deep brine (808 nmol/l) is much lower compared with the Atlantis and Kebrit brines (Schmidt et al., 2003). Our approach failed to amplify *pmoA* from DD-I or to identify any methanotrophic-assigned OTUs in the 16S rRNA library, though one methylotrophic OTU was identified in DD-I (Figure 3A). These results are in accord with a recent study by Bougouffa and coworkers where aerobic methanotrophs were not detected in DD-I (Bougouffa et al., 2013). However, the protein-based phylogenetic analysis of our DD-I shotgun pyrosequencing metagenomic data assigned reads to methanotrophs from genera including *Methylococcus, Methylacidiphilum, Methylocella, Methylosinus, Methylocystis*, and *Methylobacter*. The methanotrophs represented 0.615% of the total significant metagenomic reads (Table 3). Our study addresses only a fraction of the aerobic methanotrophs because the use of the 0.1 μm filter may have eliminated some aerobic methanotroph species, such as the genus *Methylomonas* (Dworkin et al., 2006).

Geochemical carbon isotope analyses of the Kebrit brine-seawater interface point to the existence of aerobic methane oxidation (Faber et al., 1998; Antunes et al., 2011b). The microbial community living at the Kebrit brine-seawater interface was previously investigated by Eder et al. (2001), and their results did not indicate the presence of any type of methane-oxidizing bacteria (Eder et al., 2001). In the present study, both the 16S rRNA and the *pmoA* gene analyses indicated the presence of aerobic methanotrophs in the Kebrit brine-seawater interface layers and that methanotrophs similar to the genus *Methylobacter* represent the main aerobic methane oxidizer in these layers (Table 3, Figure 4A). Phylogenetic analysis of 16S rRNA OTUs revealed taxa that are closely related to uncultured aerobic methanotrophs from diverse marine environments as well as cultured members of the genus *Methylobacter* (Figure 3A). KB-U exhibited the greatest number of metagenomic reads obtained through shotgun pyrosequencing that were assigned to methanotrophs in our study. The *pmoA* phylotypes showed that the diversity of aerobic methanotrophs in KB-U was lower than in ATII-I and was mainly dominated by methanotrophs belonging to the genus *Methylobacter* (Table 3, Suplementary Table 4). Similar to the samples from DD-I, no *pmoA* sequences were amplified from KB-L. Although KB-L exhibits a higher concentration of methane, the methanotroph abundance in this interface was shown to be very low (~0.1%) according to the 16S rRNA analysis (0.34%) based on total significant shotgun pyrosequencing metagenomic reads, and methanotrophs were undetectable in the *pmoA* library. It was previously proposed that a high hydrogen sulfide concentration does not favor the metabolism of aerobic methanotrophs and may therefore account for their low abundance/absence in the H_2_S-rich KB-L (Hartmann et al., 1998; Schmidt et al., 2003).

DNA sequences encoding homologs of protein subunits of known enzymes involved in methane metabolism were detected and were similar in the KB-U and KB-L layers, which presented sequences that were distinct from those found in ATII-I and DD-I. These findings are demonstrated in the NMDS analysis (Figure 5) and in the hierarchical clustering of putative enzymes involved in methane metabolism (Supplementary Figure 1 and Table 5). The distinct pattern observed in the NMDS analysis through the ordination of putative enzymes involved in methane metabolism may not apply to other enzymatic pathways and therefore only indicates the distribution of microbes involved in methane metabolism.

The metabolic reconstruction of the putative methane metabolism pathways present in some of the obtained microbial species was based on comparing the four metagenomic libraries against the KEGG database, and the results implied that the two Kebrit layers share similar methane metabolism-associated enzymes. The number of reads associated with methane metabolism was highest in KB-U, followed by KB-L and DD-I and, finally, ATII-I. The detection of DNA sequences that were homologous to different subunits of the key enzyme methane monooxygenase and other enzymes involved in methane oxidation in KB-U indicates a higher abundance of aerobic methanotrophs in this layer than in all of the other layers studied, which is in agreement with the 16S rRNA and protein-based phylogenetic analyses.

We primarily detected type I methanotrophs using both 16S rRNA and *pmoA* libraries. However, the obtained shotgun pyrosequencing metagenomic reads showed hits to type II methanotrophs such as *Methylocella*, *Methylocystis, Methylosinus, and Methylocapsa* in all four libraries (Table 3). Additionally, reads presenting hits to *Methylacidiphilum* (Verrucomicrobia) were also detected in all four libraries (Table 3). Shotgun pyrosequencing metagenomic reads encoding sMMO homologs were identified in KB-U, KB-L, ATII-I, and DD-I. It should be noted that pMMO homologs were only identified KB-U (Table 4).

Our results indicated that despite the low abundance of the aerobic methanotroph community in the Atlantis II Deep, Discovery Deep and Kebrit Deep brine interfaces, the diversity and novelty of the aerobic methanotrophs present, particularly in ATII-I, may play a key role in methane metabolism. Our results provide additional information that could contribute to our understanding of the microbial communities existing at these “anoxic-oxic” and “brine-seawater” boundaries. Future studies on the novel aerobic methanotrophic bacteria found at ATII-I and aimed at biochemical characterization of the methane monooxygenase enzymes found in Red Sea brine pools would add to our understanding on any potential novel substrate specificity.

### Conflict of interest statement

The authors declare that the research was conducted in the absence of any commercial or financial relationships that could be construed as a potential conflict of interest.
