# Supplementary material for: Aerobic methanotrophic communities at the Red Sea brine-seawater interface
Source: Front Microbiol. 2014 Sep 23;5:487. doi: 10.3389/fmicb.2014.00487 (PMC4172156; doi:10.3389/fmicb.2014.00487)
Supplement: Supplementary file 1 [file Presentation1.PDF]

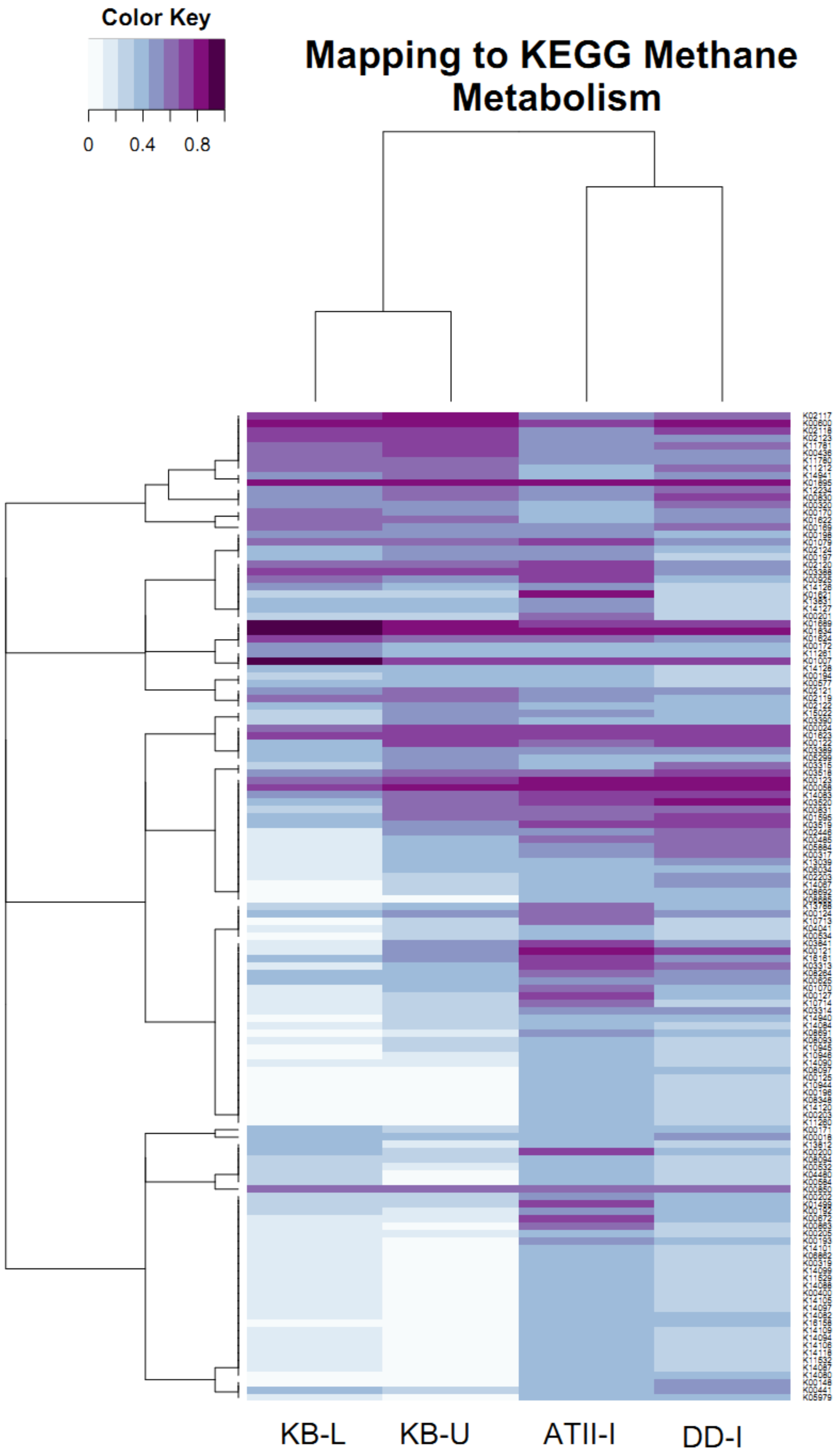
Color Key
0
0.4
0.8
Mapping to KEGG Methane Metabolism
KB-L
KB-U
ATII-I
DD-I

Supplementary Figure 1- Heat map for KEGG Mapping to Methane Metabolism

Heat map for reads abundance counts recruited to different KEGG orthologous groups and belonging to methane metabolism pathway across AT-I, DD-I, KB-UI and KB-L. Frequencies were normalized, log2 transformed, ordered by heirarchial clustering (Spearman correlation matrix with complete linkage) and scaled.
