# Supplementary material for: Aerobic methanotrophic communities at the Red Sea brine-seawater interface
Source: Front Microbiol. 2014 Sep 23;5:487. doi: 10.3389/fmicb.2014.00487 (PMC4172156; doi:10.3389/fmicb.2014.00487)
Supplement: Supplementary file 2 [file DataSheet1.PDF]

Supplementary Table 1- 16S rRNA interface unique only pyrotags

Samples	Total pyrotags	Interface unique pyrotags *
**ATII-I**	16618	13357
**DD-I**	16457	3407
**KB-U**	13904	8234
**KB-L**	13523	10981

* After subtraction from ATII water column (97% identity cutoff) and statistical filtering using Fisher exact test
