# Supplementary material for: Aerobic methanotrophic communities at the Red Sea brine-seawater interface
Source: Front Microbiol. 2014 Sep 23;5:487. doi: 10.3389/fmicb.2014.00487 (PMC4172156; doi:10.3389/fmicb.2014.00487)
Supplement: Supplementary file 3 [file DataSheet2.PDF]

Supplementary Table 2- Alpha diversity indices for both 16 S rRNA and *pmoA* libraries

Samples	Libraries	Number of clones	Number of phylotypes/OTUs	Chao1	Shannon	Simpson's index
**ATII-I**	*pmoA*	59	6	6	1.3	0.62
	16S rRNA	10636	4756	6654.9	7.1	0.89
**DD-I**	16S rRNA	15383	11955	33876.96	12.2	0.99
**KB-U**	*pmoA*	80	2	2	0.12	0.05
	16S rRNA	12017	8496	36316.9	10.6	0.98
**KB-L**	16S rRNA	12105	9192	21520.3	10.5	0.99
