# Supplementary material for: Aerobic methanotrophic communities at the Red Sea brine-seawater interface
Source: Front Microbiol. 2014 Sep 23;5:487. doi: 10.3389/fmicb.2014.00487 (PMC4172156; doi:10.3389/fmicb.2014.00487)
Supplement: Supplementary file 4 [file DataSheet3.PDF]

Supplementary Table 3- Assignment of brine seawater interface unique 16S rRNA to bacterial taxonomic groups

Taxonomical groups *	Unique ATII-I	Unique DD-I	Unique KB-U	Unique KB-L
**Marine Group I**	0.09%	4.67%	6.53%	6.48%
**Deep Sea Euryarcheotic Group**	0.00%	0.00%	0.04%	0.20%
**Halobacteriaceae**	0.00%	0.26%	0.00%	0.00%
**Miscellaneous Euryarcheotic Group**	0.00%	0.09%	0.12%	0.28%
**Marine Benthic Group_E**	0.16%	0.00%	0.00%	0.52%
**South African Goldmine Group**	0.01%	1.44%	0.00%	0.15%
**Acidobacteriaceae**	0.00%	0.47%	0.01%	0.05%
Iamia	0.01%	0.26%	1.36%	0.13%
**Acidimicrobiales**	0.04%	0.18%	0.39%	0.01%
Georgenia	0.00%	0.50%	0.00%	0.00%
Brevibacterium	0.00%	0.70%	0.00%	0.00%
Brachybacterium	0.00%	0.29%	0.00%	0.00%
Cellulosimicrobium	0.00%	0.29%	0.00%	0.00%
**Marinilabiaceae**	0.00%	0.00%	0.00%	0.27%
Owenweeksia	0.00%	0.00%	0.28%	0.04%
**Sphingobacteriaceae**	0.00%	0.29%	0.00%	0.00%
Simkania	0.00%	0.00%	0.01%	0.18%
**Dehalogenimonas**	0.04%	0.00%	0.05%	0.37%
**SAR202**	0.08%	2.96%	0.23%	0.00%
**Cyanobacteria**	0.00%	0.32%	0.00%	0.00%
**SAR406**	12.73%	6.34%	27.42%	4.42%
**Deferribacterales**	0.00%	0.79%	0.07%	0.01%
Virgibacillus picturae	0.00%	0.23%	0.00%	0.00%
Paenibacillus	0.00%	0.41%	0.00%	0.00%
Fusibacter	0.00%	0.00%	0.00%	0.13%
**Syntrophomonadaceae**	0.00%	0.00%	0.02%	0.18%
Fusobacteria	0.01%	0.00%	0.02%	0.23%
**Gemmatimonadetes**	0.00%	0.82%	0.04%	0.06%
Nitrospira	0.00%	2.55%	0.02%	0.00%
**OD1**	0.19%	1.97%	7.04%	25.83%
**OP11**	0.00%	0.00%	0.00%	0.19%
**OP1**	0.01%	0.00%	0.07%	1.33%
**OP3**	0.02%	0.00%	1.97%	14.07%
**OP8**	0.00%	0.00%	0.04%	0.27%
**Phycisphaeraceae**	0.00%	0.09%	0.13%	0.16%
**Phycisphaerae**	0.00%	0.00%	0.04%	0.56%

**Planctomycetaceae**	0.00%	0.03%	0.26%	0.11%
Candidatus scalindua	1.55%	0.00%	14.70%	0.33%
Brevundimonas	0.01%	1.73%	0.00%	0.00%
Devosia	0.00%	0.23%	0.00%	0.00%
Aminobacter	0.01%	0.26%	0.00%	0.00%
Phyllobacterium myrsinacearum	10.24%	0.03%	0.00%	0.00%
Phyllobacterium	53.99%	0.09%	0.00%	0.00%
Ensifer	0.00%	2.05%	0.00%	0.00%
Rhizobium	0.00%	0.76%	0.00%	0.00%
**Rhodobacteraceae**	0.03%	0.03%	0.04%	0.72%
Defluviicoccus	0.01%	0.21%	0.33%	0.07%
**Rhodospirillaceae**	0.07%	3.55%	2.00%	0.42%
**Rhodospirillales**	0.00%	0.23%	0.00%	0.00%
Pelagibacter	0.00%	21.40%	0.83%	0.02%
**SAR11**	0.01%	0.79%	0.21%	0.01%
Rickettsiales	0.00%	3.35%	0.16%	0.05%
**Alphaproteobacteria**	0.00%	0.47%	0.06%	0.01%
Achromobacter	0.00%	2.29%	0.00%	0.00%
Bordetella	0.00%	0.21%	0.00%	0.00%
Cupriavidus	1.45%	0.06%	0.00%	0.00%
Limnobacter	0.00%	0.00%	0.01%	0.29%
Ralstonia	0.28%	0.09%	0.00%	0.01%
Acidovorax	0.00%	0.00%	0.00%	0.56%
**Bacteriovoraceae**	0.01%	0.18%	0.00%	0.19%
**Desulfarculaceae**	0.01%	0.00%	0.43%	0.35%
**Desulfobacteraceae**	0.01%	0.00%	2.11%	2.97%
Nitrospina	0.27%	0.21%	4.15%	0.08%
Myxococcus	0.00%	3.05%	0.00%	0.00%
**Myxococcales**	0.00%	0.73%	0.01%	0.03%
**SAR324**	0.01%	3.29%	1.70%	0.01%
**Deltaproteobacteria**	0.11%	0.50%	0.84%	1.66%
Sulfurimonas	0.17%	0.00%	0.56%	0.18%
Sulfurovum	0.00%	0.00%	0.70%	2.51%
Alteromonas	0.11%	2.29%	0.00%	0.01%
Teredinibacter	0.00%	0.26%	0.00%	0.00%
Idiomarina	0.01%	0.94%	0.00%	0.01%
Coxiella	0.00%	0.91%	0.02%	0.08%
**Legionellaceae**	0.00%	0.03%	0.02%	0.25%
Methylobacter luteus	0.00%	0.00%	1.87%	0.05%
Methylobacter	0.00%	0.00%	1.03%	0.05%
Halomonas	0.01%	0.47%	0.00%	0.02%

Marinomonas	0.01%	0.32%	0.00%	0.00%
Oceanobacter	0.00%	0.41%	0.00%	0.00%
Pseudospirillum	0.00%	0.26%	0.06%	0.00%
**SAR86**	0.00%	1.67%	0.01%	0.00%
**Oceanospirillales**	0.05%	0.47%	0.04%	0.00%
Acinetobacter	0.01%	0.03%	0.01%	0.27%
Enhydrobacter	16.90%	0.00%	0.00%	0.00%
Pseudomonas putida	0.00%	0.23%	0.00%	0.00%
Pseudomonas	0.02%	0.91%	0.00%	0.05%
**Salinisphaeraceae**	0.00%	2.08%	0.02%	0.00%
Mariprofundus	0.88%	0.00%	5.13%	0.10%
aminisulfidivoran	0.00%	0.00%	1.46%	0.30%
Methylophaga	0.00%	0.00%	0.85%	0.05%
Piscirickettsia	0.00%	0.35%	0.00%	0.00%
Pyura chilensis	0.00%	0.00%	0.02%	0.52%
**Thiomicrospira**	0.00%	0.00%	0.02%	0.34%
Leucothrix	0.21%	0.00%	0.00%	0.00%
**Sinobacteraceae**	0.00%	0.44%	0.27%	0.08%
**Stenotrophomonas**	0.00%	0.70%	0.00%	0.00%
**Gammaproteobacteria**	0.00%	0.88%	0.78%	0.38%
**Spirochaeta**	0.00%	0.00%	0.23%	0.36%
**Spirochaetes**	0.00%	0.00%	0.44%	1.45%
**TM6**	0.01%	0.12%	0.07%	2.09%
**WS3**	0.01%	0.09%	0.04%	1.77%
**Bacteria****	0.09%	0.29%	5.02%	20.50%
**Unknown*****	0.03%	14.06%	7.58%	4.56%

*Based on VAMPS taxonomical classification after removing 16S rRNA similar to ATII water column and statically filtered through fisher exact test

**Bacteria = pyrotags that are assigned to bacterial origin

***Unknown = pyrotags that are assigned to unknown origin
