# Supplementary material for: Aerobic methanotrophic communities at the Red Sea brine-seawater interface
Source: Front Microbiol. 2014 Sep 23;5:487. doi: 10.3389/fmicb.2014.00487 (PMC4172156; doi:10.3389/fmicb.2014.00487)
Supplement: Supplementary file 5 [file DataSheet4.PDF]

Supplementary Table 4- Assignment of *pmoA* clones to the closest cultured and uncultured methanotrophs

	Uncultured Methanotrophs		Cultured methanotrophs	
**Clone name**	Description	Fraction Identical (%)	Description	Fraction Identical (%)
**AT-A01**	ABY90350-PmoA [uncultured type I methanotroph]	98.21	YP_115248-methane monooxygenase subunit A [Methylococcus capsulatus str. Bath]	88.69
**AT-A02**	Not Available	-	BAH22842-methane monooxygenase protein A [Methylomarinum vadi]	97.08
**AT-A03**	ABY90350P-moA [uncultured type I methanotroph]	98.21	YP_115248-methane monooxygenase subunit A [Methylococcus capsulatus str. Bath]	88.69
**AT-A04**	AGO87376-particulate methane monooxygenase, partial [uncultured bacterium]	88.17	YP_115248-methane monooxygenase subunit A [Methylococcus capsulatus str. Bath]	83.43
**AT-A05**	ABY90350P-moA [uncultured type I methanotroph]	98.73	AAQ10311-particulate methane monooxygenase [Methylococcus capsulatus]	88.54
**AT-A06**	AGO87376-particulate methane monooxygenase, partial [uncultured bacterium]	87.57	YP_115248-methane monooxygenase subunit A [Methylococcus capsulatus str. Bath]	82.84
**AT-A07**	AGO87376-particulate methane monooxygenase, partial [uncultured bacterium]	87.2	YP_115248-methane monooxygenase subunit A [Methylococcus capsulatus str. Bath]	82.32
**AT-A08**	ABY90350-PmoA [uncultured type I methanotroph]	95.3	YP_115248-methane monooxygenase subunit A [Methylococcus capsulatus str. Bath]	86.58
**AT-A09**	ABY90350-PmoA [uncultured type I methanotroph]	98.21	YP_115248-methane monooxygenase subunit A [Methylococcus capsulatus str. Bath]	88.69
**AT-A10**	ABY90350-PmoA [uncultured type I methanotroph]	98.21	YP_115248-methane monooxygenase subunit A [Methylococcus capsulatus str. Bath]	88.69
**AT-B02**	AGO87376-particulate methane monooxygenase, partial [uncultured bacterium]	87.43	YP_115248-methane monooxygenase subunit A [Methylococcus capsulatus str. Bath]	82.63
**AT-B03**	AGO87376-particulate methane monooxygenase, partial [uncultured bacterium]	87.57	YP_115248-methane monooxygenase subunit A [Methylococcus capsulatus str. Bath]	82.84
**AT-B04**	AGO87376-particulate methane monooxygenase, partial [uncultured bacterium]	87.57	YP_115248-methane monooxygenase subunit A [Methylococcus capsulatus str. Bath]	82.84
**AT-B05**	ABY90350-PmoA [uncultured type I methanotroph]	98.21	YP_115248-methane monooxygenase subunit A [Methylococcus capsulatus str. Bath]	88.69
**AT-B06**	Not Available	-	BAH22842-methane monooxygenase protein A [Methylomarinum vadi]	97.5
**AT-B09**	Not Available	-	BAF62077-particulate methane monooxygenase protein A [Methylomarinum vadi]	97.01
**AT-B10**	ABY90350-PmoA [uncultured type I methanotroph]	97.62	YP_115248-methane monooxygenase subunit A [Methylococcus capsulatus str. Bath]	86.51
**AT-B11**	ABY90350-PmoA [uncultured type I methanotroph]	97.62	YP_115248-methane monooxygenase subunit A [Methylococcus capsulatus str. Bath]	86.51

**AT-B12**	ABY90350-PmoA [uncultured type I methanotroph]	97.62	YP_115248-methane monooxygenase subunit A [Methylococcus capsulatus str. Bath]	88.1
**AT-C02**	ABC86668-particulate methane monooxygenase [uncultured gamma proteobacterium]	65.34	AAB49821-particulate methane monooxygenase 27 kDa subunit [Methylococcus capsulatus str. Bath]	63.07
**AT-C03**	ACC85993-PmoA, partial [uncultured bacterium]	85.89	YP_115248-methane monooxygenase subunit A [Methylococcus capsulatus str. Bath]	82.82
**AT-C05**	ABY90350-PmoA [uncultured type I methanotroph]	97.04	YP_115248-methane monooxygenase subunit A [Methylococcus capsulatus str. Bath]	87.57
**AT-C06**	AGO87376-particulate methane monooxygenase, partial [uncultured bacterium]	87.95	YP_115248-methane monooxygenase subunit A [Methylococcus capsulatus str. Bath]	83.13
**AT-C07**	AGO87376-particulate methane monooxygenase, partial [uncultured bacterium]	87.88	YP_115248-methane monooxygenase subunit A [Methylococcus capsulatus str. Bath]	83.03
**AT-C08**	ACC85993-PmoA, partial [uncultured bacterium]	84.87	YP_115248-methane monooxygenase subunit A [Methylococcus capsulatus str. Bath]	81.58
**AT-C09**	ABY90350-PmoA [uncultured type I methanotroph]	98.2	YP_115248-methane monooxygenase subunit A [Methylococcus capsulatus str. Bath]	88.02
**AT-C10**	Not Available	-	BAF62077-particulate methane monooxygenase protein A [Methylomarinum vadi]	97.04
**AT-C11**	ABY90350-PmoA [uncultured type I methanotroph]	98.22	YP_115248-methane monooxygenase subunit A [Methylococcus capsulatus str. Bath]	88.24
**AT-C12**	ABY90350-PmoA [uncultured type I methanotroph]	97.04	YP_115248-methane monooxygenase subunit A [Methylococcus capsulatus str. Bath]	88.17
**AT-D01**	ABY90350-PmoA [uncultured type I methanotroph]	98.21	YP_115248-methane monooxygenase subunit A [Methylococcus capsulatus str. Bath]	88.69
**AT-D03**	ABY90350-PmoA [uncultured type I methanotroph]	98.2	YP_115248-methane monooxygenase subunit A [Methylococcus capsulatus str. Bath]	88.62
**AT-D05**	ABY90350-PmoA [uncultured type I methanotroph]	97.97	YP_115248-methane monooxygenase subunit A [Methylococcus capsulatus str. Bath]	87.16
**AT-D06**	ACC85993-PmoA, partial [uncultured bacterium]	85.89	YP_115248-methane monooxygenase subunit A [Methylococcus capsulatus str. Bath]	82.82
**AT-D07**	ABC86668-particulate methane monooxygenase [uncultured gamma proteobacterium]	62.15	AAB49821-particulate methane monooxygenase 27 kDa subunit [Methylococcus capsulatus str. Bath]	59.89
**AT-D08**	AGO87376-particulate methane monooxygenase, partial [uncultured bacterium]	87.57	YP_115248-methane monooxygenase subunit A [Methylococcus capsulatus str. Bath]	82.84
**AT-D09**	Not Available	-	BAH22842-methane monooxygenase protein A [Methylomarinum vadi]	95.86
**AT-D10**	ACP41280-particulate methane monooxygenase alpha subunit [uncultured bacterium]	98.82	BAL04120-particulate methane monooxygenase alpha-subunit, partial [Methylococcaceae bacterium OS501]	76.92
**AT-D12**	ABY90350-PmoA [uncultured type I methanotroph]	96.97	YP_115248-methane monooxygenase subunit A [Methylococcus capsulatus str. Bath]	87.27
**AT-E01**	ABY90350-PmoA [uncultured type I methanotroph]	95.65	YP_115248-methane monooxygenase subunit A [Methylococcus capsulatus str. Bath]	85.71

**AT-E04**	ABC86668-particulate methane monooxygenase [uncultured gamma proteobacterium]	65.54	AAB49821-particulate methane monooxygenase 27 kDa subunit [Methylococcus capsulatus str. Bath]	63.28
**AT-E05**	ABY90350P-moA [uncultured type I methanotroph]	97.62	YP_115248-methane monooxygenase subunit A [Methylococcus capsulatus str. Bath]	88.1
**AT-E06**	AGO87376-particulate methane monooxygenase, partial [uncultured bacterium]	86.67	AAQ10311-particulate methane monooxygenase [Methylococcus capsulatus]	82.67
**AT-E07**	ABY90350-PmoA [uncultured type I methanotroph]	96.97	YP_115248-methane monooxygenase subunit A [Methylococcus capsulatus str. Bath]	87.88
**AT-E08**	ABY90350-PmoA [uncultured type I methanotroph]	98.2	YP_115248-methane monooxygenase subunit A [Methylococcus capsulatus str. Bath]	88.62
**AT-E09**	ABY90350-PmoA [uncultured type I methanotroph]	97.6	YP_115248-methane monooxygenase subunit A [Methylococcus capsulatus str. Bath]	88.02
**AT-E10**	AGI16288-particulate methane monooxygenase subunit alpha, partial [uncultured bacterium]	64.02	BAM38053-putative ethylene monooxygenase protein A [Haliea sp. ETY-M]	62.2
**AT-E11**	ABY90350-PmoA [uncultured type I methanotroph]	98.15	YP_115248-methane monooxygenase subunit A [Methylococcus capsulatus str. Bath]	88.27
**AT-E12**	ACP41280-particulate methane monooxygenase alpha subunit [uncultured bacterium]	100	BAL04120-particulate methane monooxygenase alpha-subunit, partial [Methylococcaceae bacterium OS501]	78.11
**AT-F01**	ABY90350-PmoA [uncultured type I methanotroph]	97.63	YP_115248-methane monooxygenase subunit A [Methylococcus capsulatus str. Bath]	87.57
**AT-F02**	ABY90350-PmoA [uncultured type I methanotroph]	98.22	YP_115248-methane monooxygenase subunit A [Methylococcus capsulatus str. Bath]	88.76
**AT-F03**	Not Available	-	BAF62077-particulate methane monooxygenase protein A [Methylomarinum vadi]	95.86
**AT-F04**	ABY90350-PmoA [uncultured type I methanotroph]	98.2	YP_115248-methane monooxygenase subunit A [Methylococcus capsulatus str. Bath]	88.62
**AT-F05**	ABY90350-PmoA [uncultured type I methanotroph]	98.22	YP_115248-methane monooxygenase subunit A [Methylococcus capsulatus str. Bath]	88.76
**AT-F06**	AGO87376-particulate methane monooxygenase, partial [uncultured bacterium]	88.02	YP_115248-methane monooxygenase subunit A [Methylococcus capsulatus str. Bath]	83.23
**AT-F10**	ABY90350P-moA [uncultured type I methanotroph]	98.21	YP_115248-methane monooxygenase subunit A [Methylococcus capsulatus str. Bath]	88.69
**AT-F12**	ABY90350-PmoA [uncultured type I methanotroph]	98.17	YP_115248-methane monooxygenase subunit A [Methylococcus capsulatus str. Bath]	88.41
**AT-G01**	Not Available	-	BAF62077-particulate methane monooxygenase protein A [Methylomarinum vadi]	97.04
**AT-G02**	ABY90350P-moA [uncultured type I methanotroph]	98.11	YP_115248-methane monooxygenase subunit A [Methylococcus capsulatus str. Bath]	88.05
**AT-G04**	AGO87376-particulate methane monooxygenase, partial [uncultured bacterium]	88.17	YP_115248-methane monooxygenase subunit A [Methylococcus capsulatus str. Bath]	83.43

**KB.1-A02**	Not Available	-	WP_020158144-ammonia monooxygenase [Methylobacter marinus]	95.86
**KB.1-A03**	Not Available	-	WP_020158144-ammonia monooxygenase [Methylobacter marinus]	96.45
**KB.1-A04**	Not Available	-	WP_020158144-ammonia monooxygenase [Methylobacter marinus]	95.86
**KB.1-A05**	Not Available	-	WP_020158144-ammonia monooxygenase [Methylobacter marinus]	96.45
**KB.1-A06**	Not Available	-	WP_020158144-ammonia monooxygenase [Methylobacter marinus]	96.45
**KB.1-A07**	Not Available	-	WP_020158144-ammonia monooxygenase [Methylobacter marinus]	95.86
**KB.1-A09**	Not Available	-	WP_020158144-ammonia monooxygenase [Methylobacter marinus]	96.45
**KB.1-A10**	Not Available	-	WP_020158144-ammonia monooxygenase [Methylobacter marinus]	96.45
**KB.1-B01**	Not Available	-	WP_020158144-ammonia monooxygenase [Methylobacter marinus]	96.45
**KB.1-B02**	Not Available	-	WP_020158144-ammonia monooxygenase [Methylobacter marinus]	96.45
**KB.1-B03**	Not Available	-	WP_020158144-ammonia monooxygenase [Methylobacter marinus]	96.45
**KB.1-B05**	Not Available	-	WP_020158144-ammonia monooxygenase [Methylobacter marinus]	96.45
**KB.1-B06**	Not Available	-	WP_020158144-ammonia monooxygenase [Methylobacter marinus]	96.45
**KB.1-B07**	Not Available	-	WP_020158144-ammonia monooxygenase [Methylobacter marinus]	95.86
**KB.1-B08**	Not Available	-	WP_020158144-ammonia monooxygenase [Methylobacter marinus]	96.45
**KB.1-B09**	Not Available	-	WP_020158144-ammonia monooxygenase [Methylobacter marinus]	96.45
**KB.1-B12**	Not Available	-	WP_020158144-ammonia monooxygenase [Methylobacter marinus]	96.45
**KB.1-C02**	Not Available	-	WP_020158144-ammonia monooxygenase [Methylobacter marinus]	96.45
**KB.1-C03**	Not Available	-	WP_020158144-ammonia monooxygenase [Methylobacter marinus]	95.86
**KB.1-C04**	Not Available	-	WP_020158144-ammonia monooxygenase [Methylobacter marinus]	95.86

**KB.1-C06**	Not Available	-	WP_020158144-ammonia monooxygenase [Methylobacter marinus]	96.45
**KB.1-C07**	Not Available	-	WP_020158144-ammonia monooxygenase [Methylobacter marinus]	96.45
**KB.1-C08**	Not Available	-	WP_020158144-ammonia monooxygenase [Methylobacter marinus]	95.86
**KB.1-C09**	Not Available	-	WP_020158144-ammonia monooxygenase [Methylobacter marinus]	95.86
**KB.1-C12**	Not Available	-	WP_020158144-ammonia monooxygenase [Methylobacter marinus]	96.45
**KB.1-D01**	Not Available	-	WP_020158144-ammonia monooxygenase [Methylobacter marinus]	96.41
**KB.1-D02**	Not Available	-	WP_020158144-ammonia monooxygenase [Methylobacter marinus]	96.45
**KB.1-D04**	Not Available	-	WP_020158144-ammonia monooxygenase [Methylobacter marinus]	96.45
**KB.1-D05**	Not Available	-	WP_020158144-ammonia monooxygenase [Methylobacter marinus]	96.45
**KB.1-D06**	Not Available	-	WP_020158144-ammonia monooxygenase [Methylobacter marinus]	96.64
**KB.1-D07**	Not Available	-	WP_020158144-ammonia monooxygenase [Methylobacter marinus]	96.45
**KB.1-D08**	Not Available	-	WP_020158144-ammonia monooxygenase [Methylobacter marinus]	96.45
**KB.1-D09**	Not Available	-	WP_020158144-ammonia monooxygenase [Methylobacter marinus]	95.86
**KB.1-D10**	Not Available	-	WP_020158144-ammonia monooxygenase [Methylobacter marinus]	96.45
**KB.1-D11**	Not Available	-	WP_020158144-ammonia monooxygenase [Methylobacter marinus]	96.45
**KB.1-D12**	Not Available	-	WP_020158144-ammonia monooxygenase [Methylobacter marinus]	96.45
**KB.1-E02**	Not Available	-	WP_020158144-ammonia monooxygenase [Methylobacter marinus]	96.45
**KB.1-E03**	Not Available	-	WP_020158144-ammonia monooxygenase [Methylobacter marinus]	96.45
**KB.1-E05**	Not Available	-	WP_020158144-ammonia monooxygenase [Methylobacter marinus]	96.45
**KB.1-E06**	Not Available	-	WP_020158144-ammonia monooxygenase [Methylobacter marinus]	96.45

**KB.1-E07**	Not Available	-	WP_020158144-ammonia monooxygenase [Methylobacter marinus]	96.45
**KB.1-E08**	Not Available	-	WP_020158144-ammonia monooxygenase [Methylobacter marinus]	96.45
**KB.1-E09**	Not Available	-	WP_020158144-ammonia monooxygenase [Methylobacter marinus]	96.43
**KB.1-E10**	Not Available	-	WP_020158144-ammonia monooxygenase [Methylobacter marinus]	96.45
**KB.1-E11**	Not Available	-	WP_020158144-ammonia monooxygenase [Methylobacter marinus]	95.86
**KB.1-E12**	Not Available	-	WP_020158144-ammonia monooxygenase [Methylobacter marinus]	96.45
**KB.1-F01**	Not Available	-	WP_020158144-ammonia monooxygenase [Methylobacter marinus]	95.86
**KB.1-F02**	Not Available	-	WP_020158144-ammonia monooxygenase [Methylobacter marinus]	96.45
**KB.1-F03**	Not Available	-	WP_020158144-ammonia monooxygenase [Methylobacter marinus]	96.45
**KB.1-F04**	Not Available	-	WP_020158144-ammonia monooxygenase [Methylobacter marinus]	96.45
**KB.1-F05**	Not Available	-	WP_020158144-ammonia monooxygenase [Methylobacter marinus]	96.45
**KB.1-F06**	Not Available	-	WP_020158144-ammonia monooxygenase [Methylobacter marinus]	96.45
**KB.1-F08**	Not Available	-	WP_020158144-ammonia monooxygenase [Methylobacter marinus]	96.45
**KB.1-F09**	Not Available	-	WP_020158144-ammonia monooxygenase [Methylobacter marinus]	96.45
**KB.1-F10**	Not Available	-	WP_020158144-ammonia monooxygenase [Methylobacter marinus]	96.45
**KB.1-F11**	Not Available	-	WP_020158144-ammonia monooxygenase [Methylobacter marinus]	96.45
**KB.1-G01**	Not Available	-	WP_020158144-ammonia monooxygenase [Methylobacter marinus]	96.45
**KB.1-G02**	Not Available	-	WP_020158144-ammonia monooxygenase [Methylobacter marinus]	96.45
**KB.1-G03**	Not Available	-	WP_020158144-ammonia monooxygenase [Methylobacter marinus]	95.86
**KB.1-G05**	Not Available	-	WP_020158144-ammonia monooxygenase [Methylobacter marinus]	96.45

**KB.1-G07**	Not Available	-	WP_020158144-ammonia monooxygenase [Methylobacter marinus]	96.45
**KB.1-G08**	Not Available	-	WP_020158144-ammonia monooxygenase [Methylobacter marinus]	96.45
**KB.1-G11**	Not Available	-	WP_020158144-ammonia monooxygenase [Methylobacter marinus]	96.45
**KB.1-H01**	Not Available	-	WP_020158144-ammonia monooxygenase [Methylobacter marinus]	96.45
**KB.1-H02**	Not Available	-	WP_020158144-ammonia monooxygenase [Methylobacter marinus]	96.45
**KB.1-H03**	Not Available	-	WP_020158144-ammonia monooxygenase [Methylobacter marinus]	96.45
**KB.1-H06**	Not Available	-	WP_020158144-ammonia monooxygenase [Methylobacter marinus]	96.45
**KB.1-H07**	Not Available	-	BAF62077-particulate methane monooxygenase protein A [Methylomarinum vadi]	96.45
**KB.1-H08**	Not Available	-	WP_020158144-ammonia monooxygenase [Methylobacter marinus]	95.86
**KB.1-H09**	Not Available	-	WP_020158144-ammonia monooxygenase [Methylobacter marinus]	96.45
**KB.1-H11**	Not Available	-	WP_020158144-ammonia monooxygenase [Methylobacter marinus]	96.45
**KB.1-A01**	Not Available	-	WP_020158144-ammonia monooxygenase [Methylobacter marinus]	96.15
**KB.1-B04**	CAO98555-methane monooxygenase subunit A [uncultured methanotrophic bacterium]	97.48	WP_020158144-ammonia monooxygenase [Methylobacter marinus]	96.23
**KB.1-C05**	Not Available	-	WP_020158144-ammonia monooxygenase [Methylobacter marinus]	95.86
**KB.1-E04**	Not Available	-	WP_020158144-ammonia monooxygenase [Methylobacter marinus]	95.86
**KB.1-G12**	Not Available	-	WP_020158144-ammonia monooxygenase [Methylobacter marinus]	96.25
**KB.2-B06**	Not Available	-	BAF62077-particulate methane monooxygenase protein A [Methylomarinum vadi]	96.45
**KB.2-C03**	CAO98555-methane monooxygenase subunit A [uncultured methanotrophic bacterium]	97.53	WP_020158144-ammonia monooxygenase [Methylobacter marinus]	96.3
**KB.2-F05**	Not Available	-	WP_020158144-ammonia monooxygenase [Methylobacter marinus]	95.91
**KB.2-G09**	Not Available	-	WP_020158144-ammonia monooxygenase [Methylobacter marinus]	95.27
