# Supplementary material for: Aerobic methanotrophic communities at the Red Sea brine-seawater interface
Source: Front Microbiol. 2014 Sep 23;5:487. doi: 10.3389/fmicb.2014.00487 (PMC4172156; doi:10.3389/fmicb.2014.00487)
Supplement: Supplementary file 6 [file DataSheet5.PDF]

Supplementary Table 5- Assignment of 454 reads to enzymes involved aerobic methane oxidation from KEGG methane metabolism pathway (K00680)

Enzyme	KEGG	Pathway	ATII-I	DD-I	KB-L	KB-U
**Particulate Methane Monooxygenase**	K10944	Key enzyme/methane oxidation to Co_2	0	0	0	1
**Particulate Methane Monooxygenase**	K10945	Key enzyme/methane oxidation to Co_2	0	0	0	5
**Particulate Methane Monooxygenase**	K10946	Key enzyme/methane oxidation to Co_2	0	0	0	3
**Soluble Methane Monooxygenase**	K16158	Key enzyme/methane oxidation to Co_2	0	1	0	0
**Soluble Methane Monooxygenase**	K16161	Key enzyme/methane oxidation to Co_2	8	8	10	30
**Methanol/alcohol dehydrogenase**	-	-	-	-	-	-
**Formaldehyde dehydrogenase**	K00148	methane oxidation to Co_2	0	6	0	0
**Formate dehydrogenase**	K00122	methane oxidation to Co_2	7	34	12	247
	K00123	methane oxidation to Co_2	24	117	57	315
	K00124	methane oxidation to Co_2	4	4	13	46
	K00125	methane oxidation to Co_2	0	0	0	1
	K00127	methane oxidation to Co_2	10	3	1	10
	K05299	methane oxidation to Co_2	0	2	12	39
	K08348	methane oxidation to Co_2	0	0	0	1
	K15022	methane oxidation to Co_2	1	1	6	44
**Formaldehyde-activating enzyme**	K10713	Oxidation via tetrahydromethanopterin (Identified in M. capsulatus)	6	0	0	6
	K08685	Oxidation via tetrahydromethanopterin (Identified in M. capsulatus)	0	2	0	1
**Methylenetetrahydromethanopterin dehydrogenase**	K00319	Oxidation via tetrahydromethanopterin (Identified in M. capsulatus)	0	0	3	0
	K10714	Oxidation via tetrahydromethanopterin (Identified in M. capsulatus)	5	0	3	5
**Methenyltetrahydromethanopterin cyclohydrolase**	K01007	Oxidation via tetrahydromethanopterin (Identified in M. capsulatus)	18	30	1582	270
**Formylmethanofuran—tetrahydromethanopterin N-formyltransferase**	K00672	Oxidation via tetrahydromethanopterin (Identified in M. capsulatus)	8	1	2	2
**Formyl-methanofuran dehydrogenase**	K00200	Oxidation via tetrahydromethanopterin (Identified in M. capsulatus)	12	1	9	10
	K00201	Oxidation via tetrahydromethanopterin (Identified in M. capsulatus)	5	0	8	6

	K00202	Oxidation via tetrahydromethanopterin (Identified in M. capsulatus)	2	3	6	6
	K00203	Oxidation via tetrahydromethanopterin (Identified in M. capsulatus)	0	0	0	1
	K00205	Oxidation via tetrahydromethanopterin (Identified in M. capsulatus)	0	0	3	3
	K11260	Oxidation via tetrahydromethanopterin (Identified in M. capsulatus)	0	0	0	1
	K11261	Oxidation via tetrahydromethanopterin (Identified in M. capsulatus)	0	1	28	16
**Glycine hydroxymethyltransferase/Serine hydroxymethyltransferase**	K00600	Serine pathway (Type II methanotrophs)[a]	12	69	426	615
**Serine---glyoxylate transaminase**	K00830	Serine pathway (Type II methanotrophs)[a]	1	28	41	88
**Glycerate dehydrogenase**	K00018	Serine pathway (Type II methanotrophs)[a]	0	4	16	13
**Glycerate kinase**	-	Serine pathway (Type II methanotrophs)[a]	-	-	-	-
**Malyl-CoA synthetase/Malate thiokinase/Malate-CoA ligase**	K08692	Serine pathway (Type II methanotrophs)[a]	0	2	0	6
	K14067	Serine pathway (Type II methanotrophs)[a]	0	4	0	9
**Malyl-CoA lyase**	K08691	Serine pathway (Type II methanotrophs)[a]	1	3	0	2
**3-hexulose-6-phosphate synthase**	K08093	Ribulose Monophosphate cycle [RuMP](Type I methanotrophs)	0	0	1	5
**6-phospho-3-hexuloisomerase**	K08094	Ribulose Monophosphate cycle [RuMP](Type I methanotrophs)	0	0	6	5
	K13831	Ribulose Monophosphate cycle [RuMP](Type I methanotrophs)	1	0	21	18
**Glucose-6-phosphate isomerase**	-	Ribulose Monophosphate cycle [RuMP](Type I methanotrophs)	-	-	-	-
**Glucose-6-phosphate dehydrogenase**	-	Ribulose Monophosphate cycle [RuMP](Type I methanotrophs)	-	-	-	-
**Phosphogluconate dehydratase/6-Phosphogluconic dehydrase**	-	Ribulose Monophosphate cycle [RuMP](Type I methanotrophs)	-	-	-	-
**2-keto-3-deoxy-6-phosphogluconate aldolase**	-	Ribulose Monophosphate cycle [RuMP](Type I methanotrophs)	-	-	-	-
**Transketolase**	-	Ribulose Monophosphate cycle [RuMP](Type I methanotrophs)	-	-	-	-

**Ribulose 5-phosphate 3-epimerase**	-	Ribulose Monophosphate cycle [RuMP](Type I methanotrophs)	-	-	-	-
**Transaldolase**	-	Ribulose Monophosphate cycle [RuMP](Type I methanotrophs)	-	-	-	-
**Ribose-5-phosphate isomerase**	-	Ribulose Monophosphate cycle [RuMP](Type I methanotrophs)	-	-	-	-
	-	Ribulose Monophosphate cycle [RuMP](Type I methanotrophs)	-	-	-	-

[a] Enzymes presented in the table are only the characteristic enzymes of the formaldehyde assimilation via serine pathway other general enzymes involved in this pathway as well as other pathways (e.g. glyoxylate cycle) are not mentioned. The enzymes presented were selected based on Hanson R and Hanson T (Hanson and Hanson, 1996).
